# Genome Evolution and Innovation across the Four Major Lineages of *Cryptococcus gattii*

**DOI:** 10.1128/mBio.00868-15

**Published:** 2015-09-01

**Authors:** Rhys A. Farrer, Christopher A. Desjardins, Sharadha Sakthikumar, Sharvari Gujja, Sakina Saif, Qiandong Zeng, Yuan Chen, Kerstin Voelz, Joseph Heitman, Robin C. May, Matthew C. Fisher, Christina A. Cuomo

**Affiliations:** ^a^ Genome Sequencing and Analysis Program, Broad Institute of MIT and Harvard, Cambridge, Massachusetts, USA; ^b^ Division of Infectious Diseases, Department of Medicine, Duke University Medical Center, Durham, North Carolina, USA; ^c^ Institute of Microbiology and Infection and School of Biosciences, University of Birmingham, Birmingham, United Kingdom; ^d^ Department of Molecular Genetics and Microbiology, Duke University Medical Center, Durham, North Carolina, USA; ^e^ Department of Infectious Disease Epidemiology, Imperial College London, London, United Kingdom; University of Queensland; Institut Pasteur

## Abstract

*Cryptococcus gattii* is a fungal pathogen of humans, causing pulmonary infections in otherwise healthy hosts. To characterize genomic variation among the four major lineages of *C. gattii* (VGI, -II, -III, and -IV), we generated, annotated, and compared 16 *de novo* genome assemblies, including the first for the rarely isolated lineages VGIII and VGIV. By identifying syntenic regions across assemblies, we found 15 structural rearrangements, which were almost exclusive to the VGI-III-IV lineages. Using synteny to inform orthology prediction, we identified a core set of 87% of *C. gattii* genes present as single copies in all four lineages. Remarkably, 737 genes are variably inherited across lineages and are overrepresented for response to oxidative stress, mitochondrial import, and metal binding and transport. Specifically, VGI has an expanded set of iron-binding genes thought to be important to the virulence of *Cryptococcus*, while VGII has expansions in the stress-related heat shock proteins relative to the other lineages. We also characterized genes uniquely absent in each lineage, including a copper transporter absent from VGIV, which influences *Cryptococcus* survival during pulmonary infection and the onset of meningoencephalitis. Through inclusion of population-level data for an additional 37 isolates, we identified a new transcontinental clonal group that we name VGIIx, mitochondrial recombination between VGII and VGIII, and positive selection of multidrug transporters and the iron-sulfur protein aconitase along multiple branches of the phylogenetic tree. Our results suggest that gene expansion or contraction and positive selection have introduced substantial variation with links to mechanisms of pathogenicity across this species complex.

## IMPORTANCE

The genetic differences between phenotypically different pathogens provide clues to the underlying mechanisms of those traits and can lead to new drug targets and improved treatments for those diseases. In this paper, we compare 16 genomes belonging to four highly differentiated lineages of *Cryptococcus gattii*, which cause pulmonary infections in otherwise healthy humans and other animals. Half of these lineages have not had their genomes previously assembled and annotated. We identified 15 ancestral rearrangements in the genome and over 700 genes that are unique to one or more lineages, many of which are associated with virulence. In addition, we found evidence for recent transcontinental spread, mitochondrial genetic exchange, and positive selection in multidrug transporters. Our results suggest that gene expansion/contraction and positive selection are diversifying the mechanisms of pathogenicity across this species complex.

## INTRODUCTION

*Cryptococcus gattii* is a pathogenic yeast of humans and other animals, which causes disease in predominantly immunocompetent hosts, unlike its opportunistic sister species *Cryptococcus neoformans* (1), which primarily causes disease in immunocompromised hosts. *C. gattii* comprises four distinct lineages (var. gattii I [VGI], -II, -III, and -IV) (2) with such considerable genetic variation that they have recently been described as separate species (*C. gattii*, *C. deuterogattii*, *C. bacillisporus*, and *C. tetragattii*, respectively) (3). However, the lineages can mate and exchange genetic material; for example, a mitochondrial hybrid derived from a VGII and VGIII *in vitro* cross was recently described (4). *C. gattii* even maintains the ability to form hybrids with *C. neoformans*, e.g., VGI-VNI (5) and VGII-VNIV (6) hybrids. Although *C. gattii* is globally ubiquitous in both environmental and clinical settings, it has some geographic population structure, such as VGI predominating in Europe, VGII predominating in North and South America, and VGIV predominating in the southern countries of Africa (7). VGI and VGII are the most frequently encountered globally (~800 from a panel of 1,000 global isolates [7]) and have been found on every continent tested. Of the four *C. gattii* clades, VGII appears to be the most basal (2, 8) and may originate from a recombining population in the rainforest of northern Brazil (9).

Although all four lineages of *C. gattii* are capable of causing disease, VGI and VGII cause the majority of infections in immunocompetent hosts, while the VGIII and VGIV groups cause infections only rarely and among predominantly immunocompromised hosts. It is currently unclear if the clinical underrepresentation of VGIII and VGIV is due to differences in their ability to cause disease or to sparsity in the environment. Hypervirulent isolates belonging to the VGII group are responsible for nearly all infections in the Pacific Northwest (PNW), including the Vancouver Island outbreak (10). VGII also differs from VGI in its clinical presentation, with a higher rate of respiratory than of central nervous system (CNS) symptoms (11, 12). To date, phospholipase B (13); laccase, which catalyzes melanin synthesis (14); and urease (15) have each been implicated in the ability of *C. neoformans* to disseminate from the lung via the lymphatic system and blood to the central nervous system. However, a systematic comparison among the four lineages of *C. gattii* for the presence or absence or genetic diversity of these genes has not been performed.

VGII outbreak strains also have an enhanced ability to rapidly proliferate within host macrophages (16), where reactive oxygen species (ROS) stimulate tubular mitochondrial morphology as a protective mechanism against autophagic degradation (17). *C. gattii* is further able to protect itself from ROS and other host-imposed stresses such as iron deprivation (18) and increased CO_2_ concentrations (19) by encapsulating itself in glucuronoxylomannan, galactoxylomannan, and mannoproteins. This polysaccharide capsule provides a physical barrier that interferes with normal macrophage phagocytosis and clearance by the immune system (20). Differences in capsule size have also been reported between lineages and even between the different subclades VGIIa and VGIIb (21). Again, it is unclear if these phenotypic differences are due to the distribution or to the allelic richness of virulence determinants.

Genome sequencing for just two isolates (VGI isolate WM276 and the VGII isolate R265) revealed an abundance of diversity, including chromosome copy number variation, genomic rearrangements, and gene presence and absence polymorphisms (1). A more recent comparison of whole-genome data generated for a diverse set of isolates illustrated the increased power for phylogenetic analysis (22) and for fine-scale mapping of recombination and variation for VGII isolates (23, 24) and for VGIII isolates (25). Such lineage-specific genetic differences may factor into phenotypic differences such as disease outcome. Here, we expand on this question by generating and comparing 16 *de novo* genome assemblies representing all four known lineages of *C. gattii*, including the first fully annotated assemblies from VGIII and VGIV. We also utilize an extended panel of 53 sequenced isolates to more fully evaluate the impact of positive selection and phylogeographic patterns.

## RESULTS

### Variation of genome structure across 16 *de novo* assemblies.

**FIG 1:**
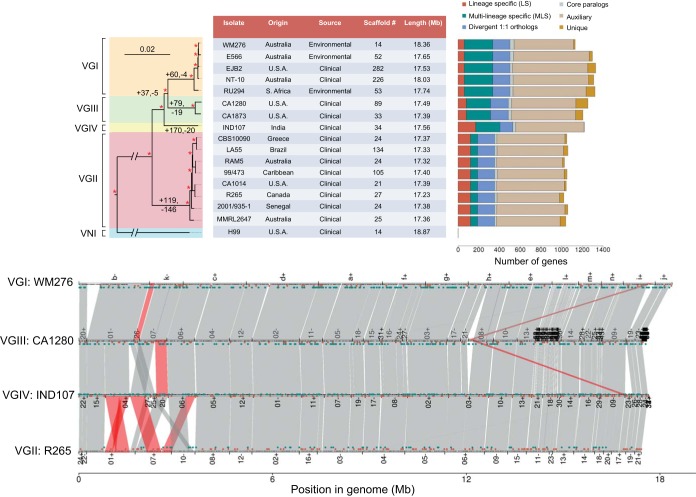
Phylogeny, gene content, and synteny of 16 *de novo* assemblies of *C. gattii*. (Top) Phylogenetic tree inferred by using RAxML from single-copy (1:1) orthologs among four lineages of *C. gattii* and the outgroup *C. neoformans*. Numbers above tree branches indicate gene gain and loss events, and asterisks indicate 100% bootstrap support from 1,000 replicates. The central table details the origin and source of each isolate, as well as the number of contigs and total length (megabases) of each assembly. The bar chart shows the numbers of lineage-specific (LS) and multilineage-specific (MLS) genes, divergent 1:1 orthologs (unclustered by OrthoMCL but identified via synteny), paralogous clusters, and auxiliary (present in ≥1 isolate but not all isolates of the encompassing lineage) and unique genes. (Bottom) Visualization of the synteny (gray) and structural variants (red) between representatives for each lineage (VGI WM276, VGII R265, VGIII CA1280, and VGIV IND107). Genes are shown as small black boxes, while LS and MLS are shown above in red and green, respectively (corresponding to the bar chart). Scaffold numbers or letters are shown along with orientation (+/−).

To explore the genomic variation among the global population of *C. gattii*, we sequenced 15 isolates representing all four known lineages and including both clinically and environmentally derived isolates (Fig. 1). Additionally, we used Illumina sequencing to make improvements to the widely used VGIIa R265α genome representing the PNW outbreak lineage. Specifically, we resolved over 124 kb of ambiguous sites (37% of previous total), replaced 4,166 single bases, and introduced 2,382 insertions and deletions. Each of the assemblies was highly contiguous and ranged in length from 17.32 to 18.36 Mb (Fig. 1). Genome length did not correlate with lineage after accounting for repetitive and low-complexity regions (see Table S1 in the supplemental material), and neither did the number of predicted and annotated protein-encoding genes, which ranged from 6,456 to 6,763. However, considerable genetic variation was identified between lineages (93% average identity and 52 single nucleotide polymorphisms [SNPs] per kb) compared with intralineage comparisons (97% average identity and fewer than 6 SNPs per kb [see Table S2 in the supplemental material).

To reconstruct the evolutionary relationships between and within the four lineages, we used 5,319 single-copy orthologous protein-coding genes from all 16 isolates, along with the widely studied *C. neoformans* VNI isolate H99 (26) as an outgroup (Fig. 1). Concordant with relationships previously determined using amplified fragment length polymorphism (AFLP) analysis (2) and sequencing of four independent genetic loci (8), we found that VGI and VGIII are the most closely related, separated from VGIV by only a short branch. VGII is the earliest-diverging and most isolated group compared to the other three. Using thousands of genes across each lineage provides robust intralineage resolution of their evolutionary relationships and their intercontinental distribution within lineages VGI and VGII.

Chromosome structure was highly conserved among the four lineages and very highly conserved within VGII. Almost all syntenic variation was identified among the three closely related lineages, VGI, VGIII, and VGIV (Fig. 1; see also Fig. S1 in the supplemental material). In total, 15 large (greater than 100-kb) chromosomal rearrangements were identified such that, on average, only 2.6% of each of the 16 genomes was rearranged with respect to the others (see Table S3 and Fig. S1). These 15 rearrangements included 10 translocations (seven interchromosomal and three intrachromosomal) and five scaffold fusions, most of which (13 of the 15) associated with clusters of predicted TcN transposons (see Fig. S1) found at centromeres (26), suggesting that these are primarily whole-chromosome arm rearrangements. Four of the rearrangements were supported by multiple isolates, including one chromosomal fusion unique to VGII (see Fig. S2), two translocations unique to VGIII (700 kb and 140 kb, respectively), and one 450-kb translocation unique to VGIV. These changes may impact the capacity for interlineage genetic exchange, as some crossover events will generate missing chromosomal regions or other aneuploidies and nonviable progeny.

In addition to chromosomal rearrangements, we identified aneuploidies in three isolates belonging to VGII and VGIII, confirmed the MAT**a** locus in all five isolates with this locus, and predicted *MAT*α in 10 previously unconfirmed isolates from all four lineages (see Table S4 in the supplemental material) using read coverage. Specifically, we found an additional (disomic) copy of scaffold 13 (SC13) in VGII veterinary isolate B8828 and a disomy of SCII in VGIII clinical isolate CA1280 (syntenic to the first half of WM276 chromosome cgba [see Fig. S2]). Variation in chromosome copy number has previously been shown to influence the virulence of *C. neoformans* (27) and can further provide resistance to azole drugs by increasing the copy number of the azole drug target (*ERG11*) or transporter (*AFR1*) commonly amplified in drug-resistant *C. neoformans* (28). However, neither gene appears to have a higher copy number in these isolates, suggesting that these aneuploidies are not associated with known drug resistance mechanisms, although they may have other effects on those isolates. We also identified a 60-kb intrachromosomal duplication in the middle of SC1 of VGII clinical isolate LA55 (also syntenic to WM276 chromosome cgba), which interestingly did not appear in the closely related isolate CBS10090. This 60-kb region covers 24 protein-encoding genes that are not known to influence drug resistance in *C. neoformans* (see Data Set S1).

### Lineage-specific genes are involved in metal ion binding, responses to oxidative stress, and mitochondrial function.

To explore the distribution of virulence-associated genes across the four lineages, we used synteny to correct all nonorthologous genes for divergent orthogroups (*n* = 175) that had not been correctly resolved (see Text S1 and Data Set S1 in the supplemental material). Key virulence genes were each found as single-copy orthologs across all four lineages, such as those that are implicated in *C. neoformans* dissemination from the lung (phospholipase B [13], laccase [*LAC1* and *LAC2*] [14], and urease [15]). In addition, at least 32 of 35 genes potentially involved in capsule biosynthesis in *C. neoformans* (29) were also single-copy orthologs in *C. gattii*. Only a UDP-glucose epimerase (CNAG_03096, Uge2) is not found in any *C. gattii* strain; mutations in a related gene, Uge1, result in larger capsule size and defective production of glucuronoxylomannogalactan (GXMGal), which is part of the capsule (30). These findings suggest that most genes involved in synthesis of the polysaccharide capsule and other genes involved in virulence are conserved between *C. gattii* and *C. neoformans*.

In total, 737 orthogroups (4,224 genes, 4.17% of all *C. gattii* genes) were lineage specific (LS) or specific to a subset of 2 or 3 lineages (multilineage specific [MLS]). These genes predominantly derived from many small intrachromosomal changes and are distributed across all chromosomes (Fig. 1; see also Text S1 and Fig. S1 in the supplemental material). By tracing the evolutionary history of these genes on our rooted phylogenetic tree, we were able to assign 661 of 737 (90%) gene clusters to a given node via a single loss or gain event (Fig. 1; Table 1; see also Data Set S1). To examine the functional significance of LS and MLS genes, we evaluated their Pfam domains and Gene Ontology (GO) terms for statistical enrichment using the two-tailed Fisher exact test with false discovery rate (FDR)-corrected *P* values (*q*) of <0.05 (see Data Set S1). We found that each lineage carries a unique subset of genes that are putatively involved in virulence and disease outcome, including genes that bind Fe^+^/Cu^+^, maintain or affect the morphology of the mitochondria, and respond to stress responses (Table 1). Furthermore, the largest enriched category of GO biological processes from all of the LS plus MLS genes combined was the response to oxidative stress.

**TABLE 1:** Top 10 significantly enriched, nonambiguous Pfam domains (*q* value, <0.05) identified across each lineage(s)

Lineage	Pfam accession no.	Pfam description^a^^,^^b^	*q* value^c^
VGI specific	PF02301.13	HORMA (HORMA domain)	3.62E−08
	PF01794.14	Ferric reduct (ferric reductase-like transmembrane component)	1.27E−06
	PF08022.7	FAD binding 8 (FAD-binding domain)	2.29E−06
	PF03151.11	TPT (triose-phosphate transporter family)	3.85E−06
	PF08030.7	NAD binding 6 (ferric reductase NAD-binding domain)	5.42E−06
	PF00098.18	zf-CCHC (zinc knuckle)	1.02E−05
	PF00628.24	PhD (PhD-finger)	3.92E−05
	PF01408.17	GFO IDH MocA (oxidoreductase family, NAD-binding Rossmann fold)	1.37E−04
	PF00005.22	ABC tran (ABC transporter)	3.02E−03
	PF04982.8	HPP (HPP family)	7.59E−03
VGI-VGIII specific	PF00098.18	zf-CCHC (zinc knuckle)	5.58E−13
	PF00160.16	Pro isomerase (cyclophilin-type peptidyl-prolyl *cis*-*trans* isomerase/CLD)	9.37E−12
VGI-VGIII lost	PF00070.22	Pyr redox (pyridine nucleotide- disulfide oxidoreductase)	8.55E−17
	PF07110.6	EthD (EthD domain)	3.04E−12
	PF07992.9	Pyr redox 2 (pyridine nucleotide- disulfide oxidoreductase)	1.17E−15
VGIII specific	PF05970.9	PIF1 (PIF1-like helicase)^a^	1.07E−02
VGIII lost	PF03952.11	Enolase N (enolase, N-terminal domain)	4.68E−43
	PF00113.17	Enolase C (enolase, C-terminal TIM barrel domain)	4.21E−42
	PF01176.14	eIF-1a (translation initiation factor 1A/IF-1)	1.68E−35
	PF07766.8	LETM1 (LETM1-like protein)^a^	1.68E−35
	PF02627.15	CMD (carboxymuconolactone decarboxylase family)	1.61E−33
	PF00732.14	GMC oxred N (GMC oxidoreductase)	3.76E−28
	PF05199.8	GMC oxred C (GMC oxidoreductase)	3.76E−28
	PF07476.6	MAAL C (methylaspartate ammonia-lyase C terminus)	1.13E−27
	PF00199.14	Catalase (catalase)	3.39E−25
	PF06628.7	Catalase-rel (catalase-related immune responsive)	3.39E−25
VGIV specific	PF13650.1	Asp protease 2 (aspartyl protease)	7.38E−03
VGIV lost	PF07883.6	Cupin 2 (Cupin domain)	1.51E−44
	PF01758.11	SBF (sodium bile acid symporter family)	1.08E−22
	PF02678.11	Pirin (Pirin)	1.08E−22
	PF00190.17	Cupin 1 (Cupin)	6.01E−22
	PF04145.10	Ctr (Ctr copper transporter family)	6.01E−22
	PF13344.1	Hydrolase 6 (haloacid dehalogenase- like hydrolase)	1.42E−20
	PF13242.1	Hydrolase-like (HAD-hydrolase-like)	1.47E−18
	PF00702.21	Hydrolase (haloacid dehalogenase-like hydrolase)	4.10E−15
	PF00631.17	G-gamma (GGL domain)	5.09E−08
VGII specific	PF04144.8	SCAMP (SCAMP family)	4.21E−16
	PF13865.1	FoP duplication (C-terminal duplication domain of Friend of PRMT1)	4.21E−16
	PF00722.16	Glyco hydro 16 (glycosyl hydrolase family 16)	2.32E−12
	PF02567.11	PhzC-PhzF (phenazine biosynthesis-like protein)	2.32E−12
	PF02893.15	Gram (GRAM domain)	2.32E−12
	PF05071.11	NDUFA12 (NADH ubiquinone oxidoreductase subunit NDUFA12)	2.32E−12
	PF00326.16	Peptidase S9 (prolyl oligopeptidase family)	9.61E−11
	PF00657.17	Lipase GDSL (GDSL-lik lipase/acylhydrolase)	9.61E−11
	PF02441.14	Flavoprotein (flavoprotein)	9.61E−11
	PF01619.13	Pro dh (proline dehydrogenase)	1.30E−10
VGII lost	PF02170.17	PAZ (PAZ domain)	1.05E−24
	PF02171.12	Piwi (Piwi domain)	1.05E−24
	PF11790.3	Glyco hydro ml (glycosyl hydrolase catalytic core)	6.10E−19
	PF01902.12	ATP bind 4 (ATP-binding region)^a^	1.13E−16
	PF00784.12	MyTH4 (MyTH4 domain)	6.24E−15
	PF02897.10	Peptidase S9 N (prolyl oligopeptidase, N-terminal beta-propeller domain)	6.24E−15
	PF08660.6	Alg14 (oligosaccharide biosynthesis protein Alg14-like)	6.24E−15
	PF12862.2	Apc5 (anaphase-promoting complex subunit 5)	6.24E−15
	PF00141.18	Peroxidase (peroxidase)	5.18E−11
	PF01713.16	Smr (smr domain)	5.18E−11

^a^ Domains belong to genes with homology to essential genes in *Saccharomyces cerevisiae*, and similar nucleotide sequence was detected in the corresponding *C. gattii* genome using tBLASTn.

^b^ Abbreviations: FAD, flavin adenine dinucleotide; HAD, haloacid dehalogenase.

^c^ Corrected *P* values were calculated from the two-tailed Fisher exact test with *q*-value FDR.

VGI has a unique expansion of genes carrying the ferric reductase-like transmembrane component and ferric reductase NAD-binding domains. Ferric reductases are involved in the production of the virulence factor melanin and resistance to azole antifungal drugs (31). Overall, VGI had the fewest LS genes of the four lineages (*n* = 60) but 12 significantly enriched Pfam domains, which also included an expansion of genes with the HORMA domain thought to be involved in DNA repair (32).

VGII isolates, which include those associated with the Vancouver Island outbreak (10), carry an expanded repertoire of secretory carrier membrane proteins (SCAMPs) involved in membrane trafficking, the Friend of Prmt1 (Fop) chromatin-associated protein domain (33), and the heat shock protein 70 (HSP70) domains found in chaperone proteins. Deletions made to the *HSP70* gene family member *Ssa1* in *C. neoformans* have indicated that *HSP70* functions as a stress-related transcriptional coactivator required for fungal virulence (34). The expansion of *HSP70* among VGII isolates suggests it as a possible mechanism for adaptation to new environments.

VGII is missing 146 genes that are present in the other three lineages, which is 3-fold fewer than corresponding gene losses in the VGI-III-IV lineages combined (Fig. 1; Table 1), further suggesting that the genomes of this lineage have been more stable over time. Enriched domains from these missing genes include the PAZ, Piwi, and DUF1785 domains, all of which are components of the RNA interference (RNAi) machinery (35), confirming previous studies that found both of the Argonaute genes *AGO1* and *AGO2* missing from previously screened VGII strains (1, 36). Further, genes lost in VGII include functional domains involved in protein processing and degradation, such as Alg14 domains, required for the second step of N-linked glycosylation (37); two S9 peptidases; and the anaphase-promoting complex subunit 5, a component of the anaphase-promoting complex. However, a translated BLAST of essential yeast genes against the VGII genomes revealed an Alg14-like sequence, suggesting that the gene may be present but that either a gene structure was not predicted or it was recently pseudogenized. Half of the PIF1-like helicases crucial for both nuclear and mitochondrial genome maintenance (38) have been lost in VGII strains; these could play a role in the different tubular mitochondrial morphology of VGII (17). In addition, peroxidases, cytochrome oxidase *c* subunit VIb (COX6B), and the ferritin iron-binding region signature 2 are also uniquely absent in the VGII isolates, each of which may be involved in the ability of *Cryptococcus* to defend itself against antioxidant stresses (39).

The inclusion of the first genomes for VGIII isolates revealed 79 LS genes that included a significant enrichment for PIF1-like helicases important for genome stability (38) and phosphopyruvate hydratases/enolases (40), which are highly conserved major fungal allergens (40). Glucose-methanol-choline (GMC) oxidoreductases, which include a number of antifungal proteins secreted by a diverse range of fungal species, were also lost in VGIII (41). Leucine zipper-EF-hand-containing transmembrane protein 1 (LETM1) was predicted in each of the other lineages but not predicted in VGIII, although a translated BLAST revealed LETM1-like sequence. Proteins with the LETM1 domain may be located in the mitochondria and involved in mitochondrial morphology (42). It is perhaps of relevance that mitochondrial hybrids have been documented from VGIII parental isolates (4).

VGIV infects predominantly immunocompromised hosts and has lost one of three genes containing the Ctr copper transporter family domains, which were present across the remaining three lineages. These genes have been shown to influence *Cryptococcus* survival during pulmonary infection and the onset of meningoencephalitis (43). Genes absent in VGIV also include Pfam domains for three of the haloacid dehalogenases (HADs), which catalyze carbon or phosphoryl group transfer reactions on a diverse range of substrates (44). VGIV also had the greatest number of LS genes (*n* = 170). However, of these, only aspartyl protease 2 was significantly enriched (Table 1).

### Lineages overlap geographically as well as show evidence for recent transcontinental spread.

To more widely examine the diversity of the *C. gattii* population, we included sequences from an additional 37 isolates, including 18 newly sequenced isolates, and identified variants from these sequences and the 16 assembled genomes (see Table S4 in the supplemental material). Representatives from all four major lineages were included; while the majority were from VGII (*n* = 31), multiple isolates represented each lineage. Most strains originated from the Pacific Northwest (PNW; *n* = 28), but multiple isolates originated from South America, Africa, Europe, Australia, and Asia. Diverse sources included clinical (*n* = 34), animal (*n* = 10), and environmental (*n* = 9) sources. We also sequenced a strain resulting from an *in vitro* interlineage cross of parent isolates CBS10090 (VGII) and NIH312 (VGIII) (4).

**FIG 2:**
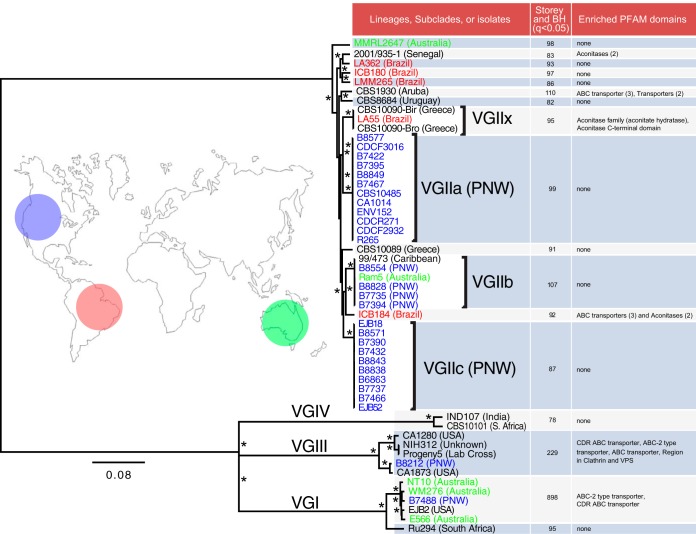
RAxML phylogeny of 53 nuclear genomes of *C. gattii*. All sites that were homozygous in all isolates and had an SNP in ≥1 isolate were used (1,432,518 sites, or 8.3% of the total length). Isolate names are colored according to geographic origin (blue, Pacific Northwest [PNW]; red, South America; green, Australia) and labeled. Isolates labeled USA are non-PNW. The asterisk indicates 100% bootstrap support using 1,000 replicates. The branch site model (BSM) of selection in Codeml was employed across 17 subclades highlighted in the table (to the right of the tree) to identify genes under selection across the internal branches within each subclade. Multiple testing correction was performed using both the Storey-Tibshirani and Benjamini-Hochberg methods (requiring *q* values of <0.05 for each). The number of genes identified as under positive selection is reported as the second column of the table. Last, enrichment Pfam domains from these genes compared with the remaining unselected genes were assessed using two-tailed Fisher’s exact test with *q*-value FDR, shown in the final column of the table.

To identify the phylogenetic relationships of these isolates, variants were identified with reference to the improved *C. gattii* VGII R265 assembly (see Table S4 in the supplemental material), and a phylogenetic tree was constructed (see Materials and Methods). The tree revealed four highly related groups within VGII (Fig. 2), representing VGIIa, VGIIb, and VGIIc and another, smaller group that we term VGIIx, which falls between VGIIa and VGIIb. Two of these groups (VGIIb and VGIIx) contained isolates from separate continents, suggesting that intercontinental transmission has occurred in recent history. VGIIb includes isolates from the PNW (*n* = 4), Australia (*n* = 1), and the Caribbean (*n* = 1). The VGIIx group includes two isolates from different continents, CBS10090 from Greece and LA55 from Brazil. These transcontinental groups, in conjunction with each of the four lineages spanning 2 or more continents, suggest recent, potentially ongoing dispersal of multiple lineages of *C. gattii*.

### Recombination between lineages is more pronounced in the mitochondrial genome than in the nuclear genome.

Phylogenetic analysis of nuclear genome-wide variation recapitulated the deeply separated VG lineages also observed in the ortholog-based phylogeny (Fig. 1). Both trees suggest that the lineages have remained largely isolated since their divergence, despite their overlapping geographic distribution and niches. Infrequent outgroup mating among the nuclear genomes of isolates is further supported by calculations of θ, Weir’s formulation of Wright’s fixation index (*F*_ST_) (45), on pairwise comparisons of each lineage using sliding windows (see Fig. S3 in the supplemental material). Across 10-kb windows of each scaffold of the nuclear genome, in each of the six pairwise comparisons (*n* = 156), values ranged between 0.77 and 0.99, suggesting that there has been little nuclear genetic exchange between the lineages. The only notable exception is a 120-kb stretch at the start of scaffold 18 in each of the pairwise comparisons (values at around 0.5). This region is where the *MAT* locus is situated, and excluding all MAT**a** isolates resulted in high *F*_ST_ values in accordance with the rest of the nuclear genome.

**FIG 3:**
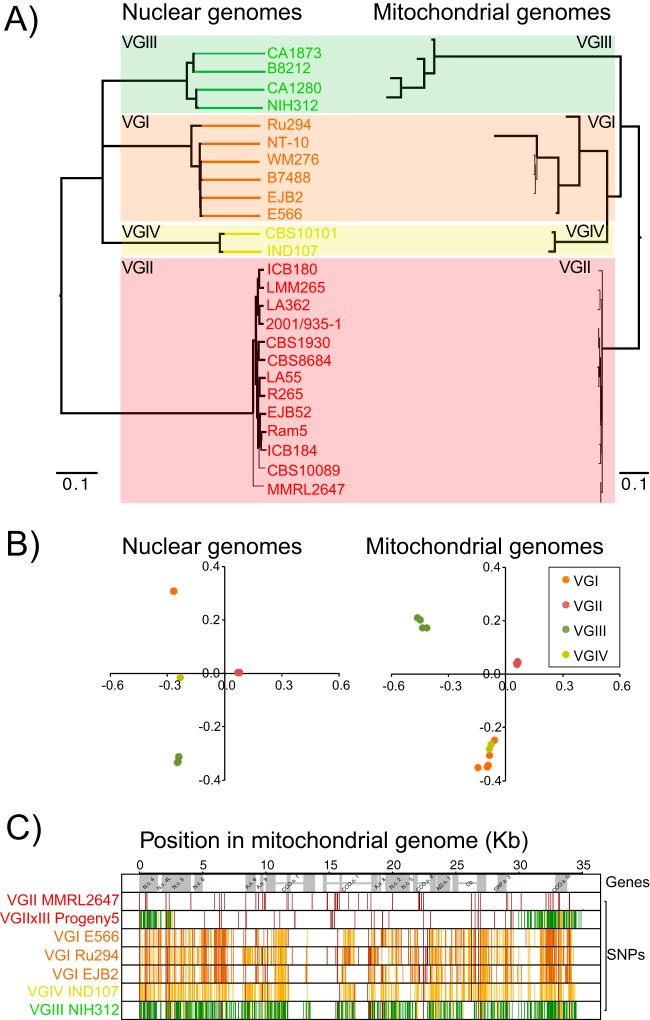
Topological discordance between the nuclear (left) and mitochondrial (right) genomes of *C. gattii*. Orange, VGI; red, VGII; green, VGIII; yellow, VGIV. (A) RAxML trees for the nuclear and mitochondrial genomes (boldface branches, 100% bootstrap support using 1,000 replicates). (B) Principal component analysis for the nuclear and mitochondrial genomes. (C) SNPs across the mitochondrial genomes of 7 isolates, including representatives from each lineage. Differences from the nuclear tree were most visible in four isolates: progeny 5, E566, Ru294, and EJB2. SNPs are colored according to the lineage to which they are unique. VGII-auxiliary (VGI-VGII, VGII-VGIII, VGII-VGIV, VGI-VGII-VGIII, VGI-VGII-VGIV, VGII-VGIII-VGIV, and VGI-VGII-VGIII-VGIV) is also colored red, while VGIV-auxiliary (VGI-VGIV, VGIII-VGIV, and VGI-VGIII-VGIV) is colored yellow. N, NADH ubiquinone oxidoreductase; ND, NADH dehydrogenase; A, ATP synthase; CCO, cytochrome *c* oxidase; Cb, cytochrome *b*; SRP, small ribosomal protein; c, chain; s, subunit.

The phylogeny estimated from nuclear site variation was next compared to that of mitochondrial genome variation. While the topologies of the mitochondrial and nuclear trees were similar, there was substantial variation in branch lengths between the two (Fig. 3A), most notably a large amount of mitochondrial diversity in VGI relative to nuclear diversity. Evaluation with principal component analysis (PCA) revealed that lineages were less discernible based on mitochondrial than on nuclear sequence (Fig. 3B), possibly in part due to recombination between these groups. Three of the six VGI isolates (E566, Ru294, and EJB2) showed greater mitochondrial sequence similarity to VGII than did their nuclear genomes. Ru294 also had a high number of shared SNPs with VGIV, including a stretch across ATP synthase subunit 6 (Fig. 3C). EJB2 and E566 also had fewer SNPs relative to VGII than did other VGI isolates (Fig. 3C). Each of these isolates has more than 100-fold depth of coverage, suggesting that this is not an artifact of lower sequencing or alignment depth. Pairwise *F*_ST_ values from VGI-VGIII (0.642), VGI-VGIV (0.672), and VGIII-VGIV (0.658) suggest that the mitochondria appear to be more recombinogenic than their nuclear counterparts (*F*_ST_ = 0.885, 0.894, and 0.885, respectively).

One of the 53 sequenced isolates was the progeny from an *in vitro* cross between parental isolates VGII CBS10090 and VGIII NIH312, previously reported (4). While the nuclear genome of one isolate of this cross (progeny 5) appeared to be almost exclusively derived from NIH312 (Fig. 3), the mitochondrial genome has inherited large regions from both parents. By overlaying the predicted genes (Fig. 3C), we found that progeny 5 had VGIII-derived copies of NADH ubiquinone oxidoreductase chain 4, 4L, and cytochrome *c* oxidase subunit III. The crossover between VGII and VGIII occurred in the middle of NADH ubiquinone oxidoreductase subunit 5, which is therefore a chimeric gene. The remaining 12 predicted genes were all from VGII, with which it clustered more closely, in contrast to its nearly VGIII-identical nuclear genome. Analysis of depth of coverage across the mitochondria identified intron loss and size variation in some isolates (see Fig. S4 in the supplemental material). For example, the intron in cytochrome *b* is also ~1 kb longer in VGII isolates than in VGI-III-IV isolates. The introns in cytochrome *c* oxidase subunits I and II are also unique to the nonbasal VGII isolates, suggesting that they have recently been acquired.

### Multidrug transporters have undergone positive selection along multiple independent branches and lineages of *C. gattii*.

To measure selection across the 53-isolate phylogeny, we employed the branch site model (BSM) of selection in Codeml, which calculates ω across genes and branches/lineages. We calculated χ^2^_1_ with multiple corrections across 17 subclades of the 53-isolate tree, measuring selection across only the terminal nodes, or recent selection (Fig. 2; see also Text S1 in the supplemental material). Between 78 and 898 genes were found to be undergoing selection in each of the 17 clades (Fig. 2; see also Data Set S1). Notable genes identified in multiple subclades include the cell wall integrity protein SCW1 in 10 subclades and iron regulator 1 in three subclades; others were unique to only one subclade, such as HSP70 in VGI, excluding Ru294.

We compared domains under selection in each of the 17 clades to all other domains (see Data Set S1 in the supplemental material). Only 20 Pfam and 2 TIGRFAM domains were significantly enriched from genes undergoing selection, and remarkably, only 8 of these were unique/nonredundant. Two domains belonging to two genes (CDR ABC transporter and ABC 2-type transporter) were independently identified in four subclades (CBS1930, ICB184, VGIII, and VGI, excluding Ru294). Additionally, the Pfam domain “ABC transporter,” belonging to a third gene, was independently enriched in three of these subclades (CBS1930, ICB184, and VGIII). Each of these transporters belongs to a single paralog cluster of six genes, which includes the ABC transporter-encoding gene *AFR1*. This class of gene includes multidrug transporters with azole and fluconazole transporter activity in *C. neoformans* (46), *Candida albicans* (47), and *Penicillium digitatum* (48). However, the closest *C. gattii* ortholog to *AFR1* was not one of the three under selection.

We also found enrichment for two iron-sulfur aconitase genes under selection in subclades 2001/935-1, VGIIx and ICB184. These genes are thought to allow *Cryptococcus* to respond to and survive nitrosative stress (49). In contrast to the major facilitator superfamily (MFS) transporters, this domain was represented by two separate orthogroups, suggesting that selection is acting on multiple gene families of similar functions. This provides two examples of selection pressures acting on similar or, indeed, identical genes relating to stress and drug transport across the phylogenetic spectrum of *C. gattii*.

## DISCUSSION

We describe for the first time a comparison of whole genomes from all four lineages of *C. gattii*. We anticipate that the sequence data, assemblies, gene predictions, and descriptions of lineage-specific features will provide a valuable resource for the community of researchers studying *Cryptococcus*. In addition to the release of these new genomes, which we primarily used to identify lineage-specific differences, we also employed a large whole-genome panel of resequenced isolates for *C. gattii* (*n* = 53), identifying high-confidence variants useful for tracking the epidemiological and evolutionary history of this species. Leveraging these data, we found multiple lineages from geographically overlapping regions, as well as evidence for recent transcontinental spread in VGIIb and the newly identified VGIIx.

While the ability for pathogens, including *C. gattii*, to avoid oxidative, nitrosative, or other host-derived stress is well described (7, 16, 39, 49), how individual strains vary in these properties is not well understood. Iron acquisition by high- and low-affinity uptake systems, as well as extracellular binding and import, is also an important virulence determinant for a number of fungal pathogens, including *Cryptococcus neoformans* (50). In this study, we used previous experimental studies on this model organism to infer the functionality of homologous genes in *C. gattii*. While this approach is a useful proxy for function in *C. gattii*, it is also unlikely to perfectly recapitulate between the two divergent *Cryptococcus* species, and may in some cases have the opposite effect or none in *C. gattii* compared with those seen in *C. neoformans*. Another issue with investigating gene loss/gain is the accuracy of gene predictions, which can miss or wrongly identify legitimate coding regions. While we have extensively evaluated our gene calls, it can be difficult in some cases to distinguish genuine disruptions to gene structures from assembly and gene prediction errors.

Despite these limitations, it is interesting that a subset of genes involved in stress response and metal acquisition in *C. neoformans* appears to be highly dynamic in terms of loss and gain by each of the four lineages and to be undergoing recent selection in *C. gattii*. While it is likely that the selection pressures driving these gene family expansions and contractions are occurring predominantly in the environment, they may result in key pathophysiological differences in humans. For example, VGI is highly ubiquitous worldwide (7) and has the fewest gene losses, which may enable it to live in a broader niche. VGII is responsible for nearly all infections in the Pacific Northwest (PNW) (10) and has the greatest number of gene losses, including (and potentially related to) those that encode the RNAi machinery (35). VGII isolates also have a large number of unique genes enriched for HSP70, COX6B, and iron-binding domains, all of which could contribute to its hypervirulence. Finally, the loss of enolases and Ctr copper transporter family domains in VGIII and VGIV, respectively, may be linked to a reduced ability of these lineages to infect immunocompetent hosts.

In addition to genes with a clear predicted role in virulence, we identified a number of genes and domains that may have a hitherto-unknown role in biological differences between the VG groups. For example, VGII isolates are uniquely enriched for Pfam SCAMP domains (33), involved in membrane trafficking, and the Fop chromatin target of protein arginine methyltransferases.

In common with most eukaryotes, cryptococcal mitochondria can shift between small punctate units and larger tubular networks of elongated mitochondria (51). However, VGII *C. gattii* strains share a unique ability to generate subpopulations with tubular mitochondria that exhibit increased intracellular proliferation within host cells (16). Both GO-term and Pfam enrichment among LS genes suggested a number of genetic differences in genes predicted to regulate the mitochondria, especially among VGII and VGIII isolates. For example, LETM1, which is involved in mitochondrial morphology in humans (42), may be absent in VGIII. VGII is missing a mitochondrial cytochrome *c* peroxidase gene, the mitochondrial import inner membrane translocase subunit TIM10, and a PIF1-like helicase, crucial for both nuclear and mitochondrial genome maintenance (38). These genes are good candidates for future work to map the genetic basis of differences in tubularization and mitochondrial morphology among the lineages.

Recently, we found that mitochondrial DNA could recombine from separate lineages when crossed in the laboratory (4). Here, we have extended our analysis by identifying a VGIIx-VGIII chimeric mitochondrial gene in progeny 5 and its contrast to the near-identical parental VGIII nuclear genome. Evidence for smaller-scale mitochondrial recombination was also found in a number of natural isolates belonging to the VGI-VGIII-VGIV lineage cluster, supported by phylogenetic methods, pairwise *F*_ST_ values, and PCA. The ability of *C. gattii* to recombine mitochondrial DNA is still poorly understood. However, it does increasingly appear that the mitochondria play important roles in disease progression and outcome (16). Indeed, in this study, we have identified a number of nuclear lineage-specific genes that respond to oxidative stress, import into the mitochondrial inner membrane, and mitochondrial maintenance or morphology. It therefore seems plausible that such differences between the nuclear genomes of each lineage are at least partly responsible for differences in the mitochondrial phenotypes.

The evolution of pathogens separated by millions of years can reveal a wide range of strategies to maintain infection or avoid detection or predation. For example, genome expansions (52), genome contractions (53), and changes in expression of effector genes (54) each contribute to maintaining a pathogen’s niche. The genome evolution of *C. gattii* shows that selection has not resulted in changes to its genome size, at least since it diverged with *C. neoformans*, but has acted across small conserved families of drug transporters and through gene expansions likely to facilitate survival and growth in the presence of an immune response. Conversely, numerous gene families have also been lost, suggesting either fluctuations in selection pressures and/or an associated cost, such as host recognition.

We show that lineage-specific virulence determinants are likely to play important roles in disease progression. However, many orthologous genes are also undergoing selection across all recently diverged subclades of *C. gattii*. Notably, a number of these genes, such as the ABC transporter *PMR5*, are independently under selection across numerous subclades (VGII ICB184, VGII CBS1930, all of VGI except Ru294, and all of VGIII). Other drug transporters under selection across multiple subclades belong to common paralogous families and may overlap in function. By resolving and making available these genomic differences, we hope to assist with untangling pathogen, host, and environmental factors, as well as providing a platform suitable for future expression, proteomic, and (ultimately) pharmacological studies.

## MATERIALS AND METHODS

### Sequencing, assembly, and annotation of 16 genomes.

Fifty-three unique isolates of *Cryptococcus gattii* were obtained from 10 countries spanning five continents and included representatives from clinical, environmental, and animal samples (see Table S4 in the supplemental material). Genomes from each of these isolates were sequenced as part of this study by one of three institutes (Birmingham University, The Broad Institute, or Imperial College London) or previously sequenced by the Translational Genomics Research Institute (22), using the Illumina HiSeq GAIIx or 2000 platform. Isolates sequenced by the CDC were obtained from the Short Read Archive (SRA) and converted from SRA format to FASTQ using the SRAtoolkit v2.3.3-4. Twenty isolates from Birmingham University/Imperial College London (BU/ICL) were recently described (4) and submitted to the SRA.

For each of the 16 new *C. gattii* genomes, genomic DNA was used to construct two libraries with average insert sizes of 197 bases and 2.5 kb as previously described (55, 56), and each library was sequenced at the Broad Institute on an Illumina HiSeq sequencer to generate 101-base paired-end reads. This sequence was assembled using ALLPATHS (57) v*R48559*. Genes were predicted and annotated by combining calls from multiple methods. A training set was generated using GeneWise and Genemark (58), and then GlimmerHmm (59), Snap (60), and Augustus (61) were used to generate *ab initio* gene models. The best gene model at a given locus was selected from these data sets using EVM (62); conserved genes missing in gene sets were identified using OrthoMCL (63) and combined with the EVM set. Genes were then filtered if >30% coding sequences (CDS) overlapped TransposonPSI (http://transposonpsi.sourceforge.net/) hits (E value, 1e−10) or overlapped repeat Pfam/TIGRFAM hits or RepeatRunner (64) proteins. RepeatModeler v1.0.7 (http://www.repeatmasker.org) was then used to identify *de novo* repeats from the assemblies. rRNAs were more completely resolved in the VGI isolates WM276 and NT10 (152 and 98 identified, respectively), compared with 5 or fewer for all other isolates, including the remaining three VGI isolates.

For R265 genome assembly improvement, *C. gattii* R265 reads were first aligned with the previous R265 genome (GenBank accession number AAFP01000000) using the Burrows-Wheeler Aligner (BWA) v0.7.4-r385 mem (65) and converted to sorted BAM format using SAMtools v0.1.9 (r783) (66). Pilon v1.5 (67) was next used to correct the assembly using these alignments, resolving 124,377 N’s (36.64% of previous total ambiguous sites), 4,166 SNPs, 936 insertions, and 1,446 deletions. While the total number of contig bases increased by 64 kb, the total scaffold length was 25,573 nucleotides (nt) smaller. For the updated R265 assembly, 5,931 of the previous 6,210 genes were mapped and 529 additional genes were added. The 279 genes that did not map from the previous R265 assembly had multiple, partially contained alignments, and all but one gene had bases with Phred quality scores of ≤25.

Genes were functionally annotated by assigning Pfam domains, GO terms, and ortholog mapping to genes of known function. HMMER3 (68) was used to identify Pfam and TIGRFAM domains, using release 27 of Pfam and release 12 of TIGRFAM. GO terms were assigned using Blast2GO (69), with a minimum E value of 1 × 10^−10^. Genes involved in capsule biosynthesis were identified based on predicted orthology to *C. neoformans* (29). Candidate missing genes were manually inspected, and in one case (*CAP64*), the gene call is partial in VGII lineages due to an assembly gap in R265.

### Ortholog-based analysis of 16 assembled genomes.

To reconstruct the evolutionary relationships between the 16 *de novo* assemblies, we identified 1:1 orthologs using OrthoMCL and generated an alignment for each gene using MUSCLE v3.8.31 (70), which was trimmed to the smallest contiguous sequence, and then all alignments were concatenated. Prottest v3.4 (79) was used to determine the best-fitting amino acid transition model according to the Bayesian information criterion (BIC). The final tree was produced using RAxML v7.7.8 (71) with 1,000 bootstrap replicates.

DAGchainer (72) was used to identify maximally scoring syntenic blocks of four or more ordered gene pairs. To identify lineage-specific genes, we corrected for divergent 1:1 orthologs using synteny (see Text S1 in the supplemental material). Multiple whole-genome alignments were built using the MULTIZ feature of the Threaded Blockset Aligner (TBA) suite of tools (73). The input dendrogram provided to MULTIZ was taken from the 16-assembly RAxML tree. The resulting pairwise alignment was projected onto WM276 to ensure that each sequence is “single coverage.” The longest alignment was also the most fragmented, which came from aligning the two largest assemblies, VGI WM276 versus VGI NT-10. The fewest mismatches (highest similarity) were found between VGIIb isolates 99/472 and RAM5, which had a 16.85-Mb match and only 757 mismatches.

### Variant calling from 53 genomes.

Each of the 53 Illumina data sets from all four research institutes was aligned with the CNB2 assembly using BWA-MEM (65) and converted to sorted BAM and Mpileup format using SAMtools54. The Genome Analysis Toolkit (GATK) (74) v2.7-4-g6f46d11 was used to call both variant and reference bases from the alignments. Briefly, the Picard tools (http://picard.sourceforge.net) AddOrReplaceReadGroups, MarkDuplicates, CreateSequenceDictionary, and ReorderSam were used to preprocess the alignments, followed by GATK RealignerTargetCreator and IndelRealigner for resolving misaligned reads close to indels. Next, GATK UnifiedGenotyper (with the haploid genotype likelihood model [GLM]) was run with both SNP and indel genotype likelihood models. We additionally ran BaseRecalibrator and PrintReads for base quality score recalibration on sites called using GLM SNP and recalled variants with UnifiedGenotyper emitting all sites. A final filtering step was used to remove any positions that were called by both GLMs (i.e., incompatible indels and SNPs).

To assess the ability of GATK v2.7-4 UnifiedGenotyper to identify variants, we realigned reads from the reference isolate R265 back with the R265 genome after introducing 60,000 SNPs (corresponding to within VGII variation) and 800,000 SNPs (maximum divergence detected) and calculated the false discovery rate (FDR) (78). For both tests, BWA-MEM aligned a greater proportion of the reads, which resulted in fewer false positives. Using the BWA-MEM alignments, GATK UnifiedGenotyper identified 99.83% true positives for the 60,000 introduced SNPs and 99.84% true positives for the 800,000 introduced SNPs. The rate of false positives was very low, with a small reduction across greater tested divergence (false-positive rate of 0.32% from 60,000; false-positive rate of 0.02% from 800,000).

### Evolutionary analysis of 53 isolates.

For phylogenetic analysis of the 53 isolates, we extracted all sites that had an SNP in ≥1 isolate and a reference or SNP in every isolate (1,432,518 sites/8.3% of the total sequence). We inferred the phylogeny of the isolates using RAxML v7.7.8 (71) with the GTRCAT model and 1,000 bootstrap replicates. Enrichment analyses were conducted using two-tailed Fisher’s exact test with *q*-value FDR. Multiple testing corrections were achieved using both the Storey-Tibshirani (75) and Benjamini-Hochberg (76) methods (requiring *q* values of <0.05 for each). For enrichment tests, we excluded Pfam, TIGRFAM, and GO terms related to transposable elements and domains of unknown function.

For selection analysis, we employed the branch site model (BSM) of selection in Codeml in PAML (77), which calculates ω across genes and branches/lineages, using a model for positive selection and a null model (ω = 1). We calculated χ^2^_1_ with concordance between both Benjamini-Hochberg (76) (*q* < 0.05) and Storey-Tibshirani (75) (*q* < 0.05) multiple corrections across 17 subclades of the 53-isolate tree, measuring selection across only the terminal nodes (recent selection).

### Accession numbers.

The 20 recent isolates from BU/ICL were submitted to the SRA under the project accession no. SRP017762. All 15 new genomes and gene calls are available in GenBank under umbrella project (PRJNA291740) and the project accession numbers ASCT01000000 (E566), ATAL01000000 (EJB2), AZGX01000000 (NT-10), ASCO01000000 (Ru294), ASCN01000000 (CA1280), ASCQ01000000 (CA1873), ATAM01000000 (IND107), AVEY01000000 (CBS10090), AZGW01000000 (LA55), ASCM01000000 (RAM5), ASCP01000000 (99/473), ASCS01000000 (CA1014), AAFP02000000 (R265), AVEX01000000 (2001/935-1), and ATAN01000000 (MMRL2647).

## SUPPLEMENTAL MATERIAL

Data Set S1 (Tab 1) Counts of initial (before syntenic corrections) lineage-specific (LS) and pan-lineage-specific (PLS) clusters and genes (from all isolates present in group) in parentheses. Genes in synteny show the total count and percentage from within the containing lineages. The most closely related isolate belonging to the following lineage was chosen to identify divergent clusters/genes that had not been identified by syntenic analysis. The final column shows the number of complementary orthogroups and genes identified by this analysis (e.g., VGII-absent genes matching VGII-lost genes). The asterisk signifies sets that have been lost twice or regained. (Tab 2) Complementary orthogroups and genes identified by syntenic orthogroups correction (green). No-longer lineage specific (LS) shows numbers of orthogroups that were reclassified as divergent orthologs. The asterisk signifies sets that have been lost twice or regained given the phylogenetic relationships of isolates. (Tab 3) Numbers of lineage-specific (LS) and multilineage-specific (MLS) genes. The first section of the table gives numbers of 1:1 ortholog and paralog clusters and genes among the containing lineages. The second section (middle 3 columns) shows the number of Pfam, TIGRFAM, and GO terms identified among each set of genes. The final 3 columns provide the number of terms that were significantly enriched compared with the remaining genes present in the containing lineages. Enrichment is determined by a two-tailed Fisher exact test with *q*-value FDR. *P* < 0.05. The asterisk signifies sets that have been lost twice or regained. (Tab 4) All enriched Pfam domains (*q* value, <0.05) of lineage-specific (LS) and multilineage-specific (MLS) genes for each of the lineages of *C. gattii*. (Tab 5) Classification of genes using Pfam and GO. Many terms were found across multiple LS plus PLS categories. For example, Cupin 1 and Cupin 2 Pfam domains were enriched among VGII-specific, VGII-lost, and VGIV-lost categories. A greater overlap of enriched GO terms was found among the LS plus PLS categories, where, for example, zinc ion binding was enriched for among VGI-specific, VGII-specific, VGII-lost, VGIII-lost, and VGI-VGII-specific categories. (Tab 6) ReviGO summary of significant GO terms from all 737 lineage-specific and pan-lineage-specific genes. Medium similarity (0.7) was used (permissive threshold to detect redundancies, *c* = 0.10). (Tab 7) Numbers of divergent genes, identified during synteny-based orthology correction. The first section of the table gives numbers of 1:1 ortholog and paralog clusters and genes among the containing lineages. The second section (middle 3 columns) shows the numbers of Pfam, TIGRFAM, and GO terms identified among each set of genes. The final 3 columns provide the number of terms that were significantly enriched compared with the remaining genes present in the containing lineages. Enrichment is determined by a two-tailed Fisher exact test with *q*-value FDR. *P* < 0.05. The asterisk signifies sets that have been lost twice or regained. (Tab 8) ReviGO summaries of significant GO terms from divergent orthologs, identified during synteny-based orthology correction. Medium similarity (0.7) was used (permissive threshold to detect redundancies, *c* = 0.10). (Tab 9) Numbers of genes (relative to R265) that have undergone selection within 17 subclades of *C. gattii* as determined by the branch site model (BSM) in Codeml. The numbers of orthologs and paralogs are shown, as are the numbers of Pfam, TIGRFAM, and GO terms identified among each set of genes. The final 3 columns provide the number of terms that were significantly enriched compared with the remaining genes present in the containing lineages. Enrichment is determined by a two-tailed Fisher exact test with *q*-value FDR. *P* < 0.05. Subsets: 1, VGIIa; 2, VGIIb; 3, ICB184; 4, CBS10089; 5, VGIIc; 6, VGIIx; 7, ICB10080; 8, LMM265; 9, LA362; 10, 2001/935-1; 11, CBS1930; 12, CBS8684, 13, MMRL2647; 14, VGI (except Ru294); 15, Ru294; 16, VGIII; 17, VGIV. Download Data Set S1, XLSX file, 0.3 MB

Text S1  Supplemental text. Download Text S1, DOC file, 0.1 MB

Figure S1  Syntenic regions across 16 *C. gattii* assemblies (azure) showing structural variants (red). DAGchainer was used to identify maximally scoring chains (minimum 4 chains) of OrthoMCL ordered gene pairs. Genes are represented as small black boxes, while locations of TCN transposons (corresponding with predicted centromeres) are shown in blue. Above the TCNs are the locations of lineage-specific (red) and multilineage-specific (green) genes represented as circles (corresponding to bar chart in Fig. 1). Supercontig numbers or letters are shown along with orientation (+/−). Download Figure S1, PDF file, 1.5 MB

Figure S2  Support of syntenic block (SB) fusion by read coverage. (A) Across region of SB2-SB3 fusion, read alignments with CA1014 were visualized using the Integrated Genomics Viewer to examine the fusion of SB2 (length, 1,124,359 bases) and SB3 identified in VGIIa CA1014, VGIIb Ram5, and MMRL2647. A drop in coverage near the fusion site is observed in all VGI, VGIII, and VGIV isolates. The single drop in VGII isolate MMRL2647 was due to a small deletion in this strain (with spanning reads). (B) Aneuploidy across the genomes was identified using the normalized depth of read coverage over each R265 supercontig for all 53 isolates and was summarized using 10-kb nonoverlapping sliding windows. Aneuploidy can be seen across supercontig 13 in VGII isolate B8828 and supercontig 11 in VGIII isolate CA1280. A large intrachromosomal duplication/expansion can also be seen in the middle of supercontig 1 for VGII isolate LA55. The start of supercontig 18 is the R265 *MAT*α locus, which is absent for the *MAT***a**-containing VGII isolates CBS10090, LA55, and CBS1930; VGI E566; and VGIII CA1873. Download Figure S2, PDF file, 0.3 MB

Figure S3  Genome-wide variation in θ, Weir’s formulation of Wright’s fixation index (*F*_ST_), on pairwise comparisons in each lineage. For comparison of isolates between each VG group, θ was calculated across window lengths of 10 kb. The lower *F*_ST_ at the start of supercontig 18 shows where the *MAT*α locus is. Below the nonoverlapping windows, mean pairwise *F*_ST_ values from all nuclear supercontigs or the mitochondrial genome are shown. Download Figure S3, PDF file, 0.1 MB

Figure S4  Average depth of read coverage over the mitochondrial genome for all 53 isolates. Isolates are ordered according to a tree constructed from variant sites from the mitochondrial sequences of all 53 isolates (aligned with R265), and gray boxes overlie each of the predicted R265 genes. Two copies of cytochrome *c* oxidase subunit 1 are predicted in R265 which are potentially absent in at least three isolates (shown by blue boxes). Depth of coverage reveals frequent intron gain/loss. Download Figure S4, PDF file, 1.2 MB

Table S1  Repeat classification in *de novo* assemblies from 16 *C. gattii* genomes. Ranges of values obtained from RepeatModeler elements detected using RepeatMasker (number of elements, length occupied [bp], and percentage of genomes) are provided for each of the four lineages. LINEs, long interspersed elements; SINEs, short interspersed elements; LTR, long terminal repeats. Table S1, PDF file, 0.02 MB

Table S2  Pairwise comparisons from each of the 16 nuclear genome assemblies from BLASTz/Threaded Blockset Aligner (TBA) alignments. For each pairwise comparison, the number of blocks, total lengths of all blocks in parentheses, the number of matches (M), the number of mismatches (MM), and the number of gaps are shown. Table S2, PDF file, 0.03 MB

Table S3  Fifteen large (>100-kb) potential chromosomal rearrangements were identified among the 16 genomes shown in Fig. S2, S3, and S4. Here, each *C. gattii* genome is divided into syntenic blocks (SB), with each row showing a discrepancy (highlighted in red), and described in the final 4 columns (the isolate, inter-/intrachromosome changes, abbreviated description, and size in kilobases). In total, these changes cover 7.3 Mb of sequence/2.6% of all the 16 genomes. hf, highly fragmented (>4 supercontigs per SB block); S, start of supercontig; E, end of supercontig; NA, nonapplicable; T, translocation; I, inversion; F, fused. *, SB9 does not have any discrepancies between these isolates but is included to complete the comparisons of each of the genomes. Table S3, PDF file, 0.05 MB

Table S4  Alignments using BWA-MEM and variant calls using GATK UnifiedGenotyper for 52 isolates of *C. gattii*. Isolates were sequenced by the Broad Institute (BI), the Centers for Disease Control and Prevention (CDC) with collaboration from Joe Heitman’s lab, or Imperial College London/Birmingham University (ICL/BU). Red signifies predicted *MAT* locus based on depth of coverage. Mb, megabase; *, *in vitro* cross between NIH312 and CBS10090. Table S4, PDF file, 0.03 MB
