# Supplementary material for: Genome Evolution and Innovation across the Four Major Lineages of Cryptococcus gattii
Source: mBio. 2015 Sep 1;6(5):e00868-15. doi: 10.1128/mBio.00868-15 (PMC4556806; doi:10.1128/mBio.00868-15)
Supplement: Text S1 — Supplemental text. Download [file mbo004152446s1.doc]

**Supplementary Text**

**Genome evolution and innovation across the four major lineages of *Cryptococcus gattii***

Rhys A. Farrer^1^, Christopher A. Desjardins^1^, Sharadha Sakthikumar^1^, Sharvari Gujja^1^, Sakina Saif^1^, Qiandong Zeng^1^, Yuan Chen^2^, Kerstin Voelz^3^, Joseph Heitman^4^, Robin C. May^3^, Matthew C. Fisher^5^, Christina A. Cuomo^1^

^1^Genome Sequencing and Analysis Program, The Broad Institute of MIT and Harvard, Cambridge, MA 02142, United States

^2^Division of Infectious Diseases, Department of Medicine, Duke University Medical Center, Durham, North Carolina, United States

^3^Institute of Microbiology and Infection & School of Biosciences, University of Birmingham, Birmingham, B15 2TT, United Kingdom

^4^Department of Molecular Genetics and Microbiology, Duke University Medical Center, 322 CARL Building, Research Drive, Box 3546, DUMC, Durham, NC 27710, United States.

^5^Department of Infectious Disease Epidemiology, Imperial College London, London, United Kingdom

**Global comparisons of 16 *C. gattii* genomes show highly contiguous assemblies and similar gene-counts.**

Genome size is mostly conserved across the four lineages. The largest assemblies were both VGI isolates (WM276 and NT-10); however, their increased size was largely explained by having the only two assemblies with predicted ribosomal RNAs (rRNA), spanning 0.4 Mb (2.22% of the genome) and 0.28 Mb (1.54%) respectively (**Table S1**). VGI isolates also had a greater abundance of Long Interspersed Elements (LINEs), unclassified repeats, and low complexity repeats compared with any other *C. gattii* lineages (28.2 kb to 115 kb additional sequences). After accounting for the repetitive and low complexity regions, differences between lineages were only small (e.g. greatest difference was an increase of VGI relative to VGII by 84 kb). To assess overall genetic variation among the isolates, each nuclear genome was aligned to one another using blastz/threaded-block aligner (TBA)(1) (**Table S2**). Inter-lineage comparisons had between 91% and 93% average identity (<52 SNPs per kb), demonstrating their considerable genetic variation compared with >97% average identity (<6 SNPs per kb) intra-lineage comparisons.

**Structural variation (inversions, translocations, and deletions)**

Across all *C. gattii* isolates, 280.81 Mb of the total 282.33 Mb (99.46%) had a syntenic block with the following, most-closely related genome. Syntenic blocks were contained within 746 of the total 1,167 supercontigs. Those 424 supercontigs without any syntenic blocks were entirely comprised of short contigs (< 25.2 kb with mean length 3.6 kb), and most (404 of the 424 total) had < 4 genes required to derive a syntenic block. Therefore, most of the contigs that appear unique to a given isolate are non-syntenic due to their size and content.

Fifteen large (>100Kb) chromosomal rearrangements were identified among the assemblies of 16 genomes of *C. gattii* based on synteny analysis (**Table S2, Fig. S2**). Five rearrangements were supported by multiple isolates. The remaining ten were each only found in a single assembly. These included end-to-end syntenic block (SB) fusions in VGII 2001/935-1, VGII MMRL2647, and VGI WM276, three translocations in WM276, two translocations in VGI Ru294 (698Kb and 187Kb), one translocation in VGI NT-10 (108Kb), and one translocation in VGIV IND107 (541Kb). Given there is no support for these three changes in any other isolate, the most conservative explanation is these events could be assembly errors. The VGII isolates were entirely syntenic with the exception of those two low-confidence variants (fusions in VGII 2001/935-1 and MMRL2647), and the fusion of SB2 to SB3 described in the main text, which may be present in all VGII isolates. The locations of TCN transposons were predicted across all 16 isolates using RepeatMasker, and all that were >30kb from another TCN transposon were excluded.

Alignments of all 53 isolates to the R265 mitochondrial sequence revealed a number of differences in intron number and length (**Fig. S5**). Firstly, the single intron of cytochrome c oxidase subunit (COXII) II (positions ~22 to 23 kb) is unique to the group of VGII isolates apart from two relatively basal VGII isolates, CBS10090 and LA55, suggesting this intron has a recent origin. One of the two introns in both copies of COXI (positions ~12 to 13 kb and 15 to 16 kb) is also unique to VGII apart from the most basal VGII isolates LA362 and MMRL2647. The second intron in one copy of Cytochrome c oxidase subunit II is also ~1.3 kb longer in VGIIa, and in a small group of VGII isolates that are not VGIIa, b or c (CBS8684, LMM265, LA362 and MMRL2647). Finally, the intron in cytochrome b is ~1 kb longer in all VGII isolates.

**Orthology-correction and lineage-specific and pan-lineage-specific genes identification**

By comparing genes clustered among the *C. gattii* isolates (without isolate H99 as an outgroup), we found 5,427 1:1 orthologs and between 194-209 in multicopy core clusters (paralogs in ≥1 isolate). A further 399-555 genes were auxiliary (genes present in ≥1 isolate, but not in all isolates of the encompassing lineage(s)). Of particular interest were the 1,076 1:1 orthogroups (including 58 in only VGI, 189 in only VGII, 113 in only VGIII and 217 in only VGIV) and eleven 1:>1 orthogroups (in total, >16% total protein coding genes per isolate) that were only present in a single lineage (lineage-specific; LS) or multiple but not all lineages (pan-lineage specific; PLS). Initial counts of genes are provided in the **Supplemental dataset**. Fifty-four LS+PLS were found on the non-syntenic supercontigs, accounting for just 0.8% of those genes, thereby discounting the non-syntenic supercontigs as a major contributor to these initial LS groups.

To correct for genes that are simply divergent rather than truly-lineage-specific, we identified the syntenic region (LS/MLS + flanking genes) for each LS/MLS gene in the most-closely related isolate in a non-harboring lineage. If the gene was present in a syntenic region (one LS/MLS gene flanked by two syntenic genes), we hypothesized that the gene was truly orthologous but had diverged to the extent that the sequence was clustered separately. In total, 379 gene clusters encompassing 3,380 non-redundant genes across the 16 isolates (48% of all LS+MLS genes) fulfilled this criterion (**Supplemental dataset**). Of these, 175 gene clusters encompassing 2,633 genes (~3% of all *C. gattii* genes; 78% of LS-MLS genes) had a syntenic partner (**Supplemental dataset**) and were no longer LS or MLS after joining orthogroups, and instead re-classified as ‘divergent 1:1 orthologs’. The remaining 580 genes were validated as LS+MLS, albeit more inclusive across the *Cg* lineages (E.g. 5 VGII-specific orthogroups became VGII-VGIV-specific).

After correcting the orthogroups by inclusion of these divergent genes, we increased the number of 1:1 orthologs from 5,427 to 5,602 (between 83% and 87% of any given *C. gattii* isolate; **Supplemental dataset**). Additionally, this analysis identified 124-167 divergent 1:1 orthologs (subset of 1:1 orthologs), 18-40 multicopy core clusters and 737 LS+MLS gene clusters (4,224 genes across all lineages) accounting for 4.17% of all *C. gattii* genes. Analysis of these synteny-corrected gene groups are described in detail in the main text.

Seventy-six MLS orthogroups (508 genes; 10% of total LS+MLS; **Supplemental dataset**) could not be placed on a single node, and instead require two loss or gain events. The simplest explanation is that they have been lost on two independent occasions, although we cannot rule out that they have been regained via recombination (perhaps inter-lineage depending on the age of origin). The most numerous sub-category are the 38 orthogroups found in only VGI and VGIV, followed by 20 orthogroups in only VGIII and VGIV, 12 in VGI and VGII, and 6 in VGII and VGIII. A total of 72 orthogroups (132 genes) were not fully syntenic, 41 of which belonged to these phylogenetically unplaced orthogroups (46 genes in total). However, 40 of these genes were in IND107, suggesting these categories derive predominantly from genomic rearrangements (and possibly recombination) along the VGIV branch.

The high degree of synteny amongst chromosomal regions (97.4%) was reciprocated among the protein-encoding genes (95% of the non-LS+MLS genes across the 16 isolates; **Supplemental dataset**). Intra-lineage synteny of LS+MLS genes was even more conserved (98%). Only 132 LS+MLS genes were not in syntenic blocks. These were from all 4 lineages and contained within 72 orthogroups. Most of these (57 orthogroups; 101 genes) had no predicted domains and were annotated ‘hypothetical protein’. The remaining orthogroups included five lost only in VGII, three of which were related to transposon activity (DDE and HNH), which have presumably translocated since the VGII diverged with VGI-III-IV. The remaining two orthogroups had the domains PF01902.12 (ATP-binding region), TIGR00290 (MJ0570_dom: MJ0570-related uncharacterized domain) and PF13668.1 (Ferritin-like domain). Other non-syntenic LS+MLS orthogroups included a phosphopyruvate hydratase lost in VGIII, a fungal specific transcription factor domain lost in VGI-VGIV, an EthD domain lost in VGI-VGIII and a Zinc knuckle lost in VGII-VGIV. VGII-VGIII had lost 4 orthogroups that were also non-syntenic, including genes with Thiolase N and C terminals, a Major Facilitator Superfamily domain, a Peptidase C14 domain, and NAD and FAD binding domains. Although we cannot say for certain if these are genuine genomic-rearrangements or errors in the assembly, none of these domains appeared in any of the complimentary ortholog groups, suggesting these orthogroups are correctly resolved.

Surprisingly, a significantly small number of LS+PLS genes were found in the chromosomal rearrangements (P<0.02, lower-tail from a *χ*² test) than the relative abundance of LS+MLS genes in syntenic regions, suggesting those rearrangements are not responsible for generating LS genes. Only 343 of the 4,224 total LS+MLS genes (8%) were found flanking one other LS+MLS gene, and 17 (0.4%) was flanked either side by LS+MLS genes. A significantly small number (29; 0.7%) of LS+MLS genes were also found at the ends of supercontigs (*p* < 7.6E-6 lower-tail from a hypergeometric distribution) – and the majority of these were from the two most fragmented assemblies. These analyses together show that C. gattii lineage-specific genes derive from many small intra-chromosomal changes rather than few large-scale changes.

By tracing the evolutionary history of these genes onto our rooted phylogenetic tree, we were able to assign 661 of 737 gene clusters (90% of total) to a given node via a single loss or gain event (**Fig. 2;** **Supplemental dataset**). VGIV had the greatest number of LS genes (n=170), followed by VGII with 119, VGIII with 79, and VGI with 60. We also examined genes absent in one of the four lineages but present in the others. VGII is missing 146 genes, which is 3-fold higher than the corresponding gene losses in the VGI-III-IV lineages combined (**Fig. 2**).

Domains enriched in genes found uniquely in VGII included all eight ribosomal L38e domains that forms part of the 60S ribosomal subunit. However, a separate 8/32 Ribosomal L7Ae are found to be uniquely absent in VGII – suggesting these are the true-counterparts. Such issues with orthology detection, even after our synteny-based corrections, may still falter given a high copy number of proteins and domains (72 in total). Enrichment for these ribosomal domains were excluded from **Fig. 2**. VGII-lost PFAM enriched domains also included two transposase related domains (DDE TNP 1 and DDE TNP 4 with *Q* < 6.24E-15 and 9.61E-23 respectively). These genes are shown to be involved in the integration/transposition of most LTR retrotransposons and many DNA transposons, which were not caught (and excluded) by the repeat-filtering step of gene-calling. These domains were therefore also excluded from **Fig. 2**.

We next performed enrichment tests for all LS+MLS genes combined against the remaining gene sets, and clustered enriched GO-terms. The largest category of biological processes was the response to oxidative stress – comprising a mitochondrial cytochrome c peroxidase lost in the VGII lineage and a catalase-related immune-responsive gene lost in the VGIII lineage. This suggests that LS+MLS genes impact the ability of different lineages to survive oxidative stresses from host macrophage phagocytosis (**Supplemental dataset**). Among the cellular component ontologies, the phosphopyruvate hydratase/enolase complex was the most abundant, while among the molecular function ontologies, zinc-ion binding was enriched in the most categories. PFAM terms were also found across multiple LS+MLS categories, including Cupin1 and Cupin2; together, 72 of a total 141 (51%) of the *C. gattii* Cupins are LS+MLS. This superfamily is extremely diverse including catalytically inactive binding proteins, as well as enzymes possessing dioxygenase, decarboxylase, and other activities (2).

Using BLASTP, we searched the final LS+MLS genes across all 16 *C. gattii* isolates against the set of all 1,156 essential genes in *S. cerevisiae* (<http://www-sequence.stanford.edu/group/yeast_deletion_project/Essential_ORFs.txt>). We found 81 top non-redundant hits (p<e-5) in total, including Alg14. We next used TBLASTN (p<e-5) to query these genes against genomes belonging to lineages they were absent from. After excluding genes with paralogs (BLASTP (p<e-5) to predicted transcripts belonging to those lineages), we identified 19 genes. For Alg14 (absent in VGII), we identified some evidence for an initial gene prediction, although this was not supported by the later stages of the pipeline and subsequently dropped. In these cases, we cannot be sure if the gene is truly present but not properly predicted, or if the gene is genuinely disrupted and therefore the absence of a prediction is correct. We have highlighted these domains in **Table 1**.

**Origin for 53 *C. gattii* isolates**

We identified variants from both the 16 isolates we assembled *de novo*, and an additional thirty-five isolates (52 in total; **Table S4**). Representatives from all four known lineages were included, with the majority from VGII (n=31), and originating from the Pacific Northwest (PNW; n=28). The Non-PNW VGII isolates were from Australia (n=2), South America (n=5), Greece (n=2, one of which was sequenced twice), the Caribbean (n=2) and Uruguay (n=1). VGI isolates were mostly from Australia (3 of 6 total), and one from South Africa. The two VGIV isolates were from a clinical source in India and a King Cheetah in South Africa. Most of the isolates were from clinical sources (30 of 51 total), including representatives from all four lineages.

**CODEML was affected by sample size on long branches of the dendrogram.**

Initially, we calculated χ^2^_1_ with concordance between both Benjamini Hochberg (q<0.05) and Storey Tibshirani (q<0.05) multiple correction. This process identified 2,564 genes under selection in VGII, 474 in VGI, 199 in VGIII and 65 in VGIV. To address if the high number of VGII genes identified was a consequence of the larger number of isolates of this lineage that were sequenced, we constructed subsets of VGII isolates with the same number of isolates in the VGI (n=6), VGIII (n=5) and VGIV (n=2) sets. This identified 1,110, 620 and 6 genes in these three VGII subsets respectively. As sample size had a dramatic effect on the outcome, we split the tree into 17 subclades, and measured selection across only the terminal nodes to detect recent selection (**Fig. 2**).

Genes undergoing selection included the cell wall integrity protein scw1 (CNBG_6143) in subclades 1, 3, 4, 5, 7, 8, 13, 14, 16 and 17, and iron regulator 1 (CNBG_9614) in subclades 1, 5 and 14 (**Fig. 2**). Others were unique to subclades, such as heat shock protein 70 (CNBG_3287) in subclade 14. Two domains (CDR ABC transporter, and ABC-2 type transporter) were independently identified in subclades 3, 11, 14 and 16. Additionally, the PFAM domain ‘ABC transporter’ was independently enriched in subclades 3, 11 and 16. All six CDR ABC transporter PFAM domains belong to a single paralog cluster of six genes, which includes the R265 gene CNBG_5107 (ABC transporter PMR5) that is under selection in all four subsets. Subclade 14 additionally has 2 other genes from this cluster (CNBG_5117; ABC transporter PMR5 and CNBG_9590; ATP-binding cassette, subfamily G (WHITE), member 2, PDR).

The TIGRFAM MFS transporter, sugar porter family domain was significantly enriched among 7 subclades (3, 4, 6, 8, 9, 11 and 14). This single enriched domain came from 8 genes within 7 orthogroups. For example, gene CNBG_4177 (predicted monosaccharide transporter) is under selection in subclades 3, 4, 6, 8, 9 and 11. This gene belongs to an orthogroup containing four other R265 genes, including CNBG_5439, which is under selection in only subclade 14. Two further genes, CNBG_4491 and CNBG_2028 belong to two separate orthogroups (each with only a single R265 gene). CNBG_4491 is under selection in subclades 4, 9 and 14, while CNBG_2028 is under selection in subclades 9 and 11. Finally, CNBG_5621 (orthogroup has 2 genes) is under selection in subclades 11 and 14, and CNBG_1635, CNBG_3009 and CNBG_5439 are all uniquely undergoing selection subclade 14. The second TIGRFAM term that was most significantly enriched along multiple branches (subclades 3, 6, 10, 14 and 16) was a mitochondrial aconitate hydratase, which was from a single gene in all 5 subsets: CNBG_2182. The final enriched TIGRFAM term was TIGR01369, carbamoyl-phosphate synthase, large subunit, which was enriched only in subclade 8.
