# Supplementary figures and images for: Genome Evolution and Innovation across the Four Major Lineages of Cryptococcus gattii
Source: mBio. 2015 Sep 1;6(5):e00868-15. doi: 10.1128/mBio.00868-15 (PMC4556806; doi:10.1128/mBio.00868-15)

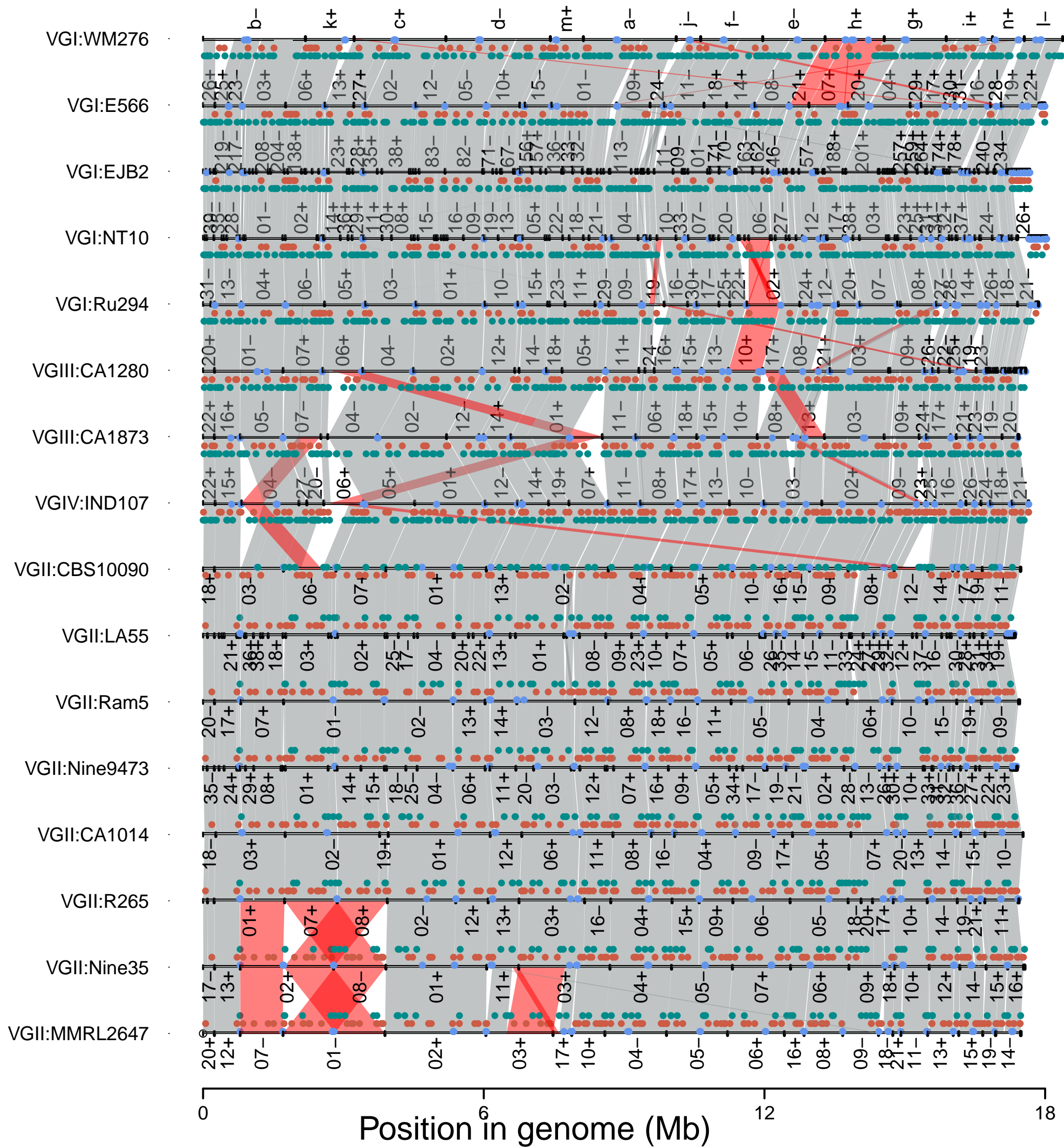

Supplement: Figure S1 — Syntenic regions across 16 C. gattii assemblies (azure) showing structural variants (red). DAGchainer was used to identify maximally scoring chains (minimum 4 chains) of OrthoMCL ordered gene pairs. Genes are represented as small black boxes, while locations of TCN transposons (corresponding with predicted centromeres) are shown in blue. Above the TCNs are the locations of lineage-specific (red) and multilineage-specific (green) genes represented as circles (corresponding to bar chart in Fig. 1). Supercontig numbers or letters are shown along with orientation (+/−). Download [file mbo004152446sf1.pdf]

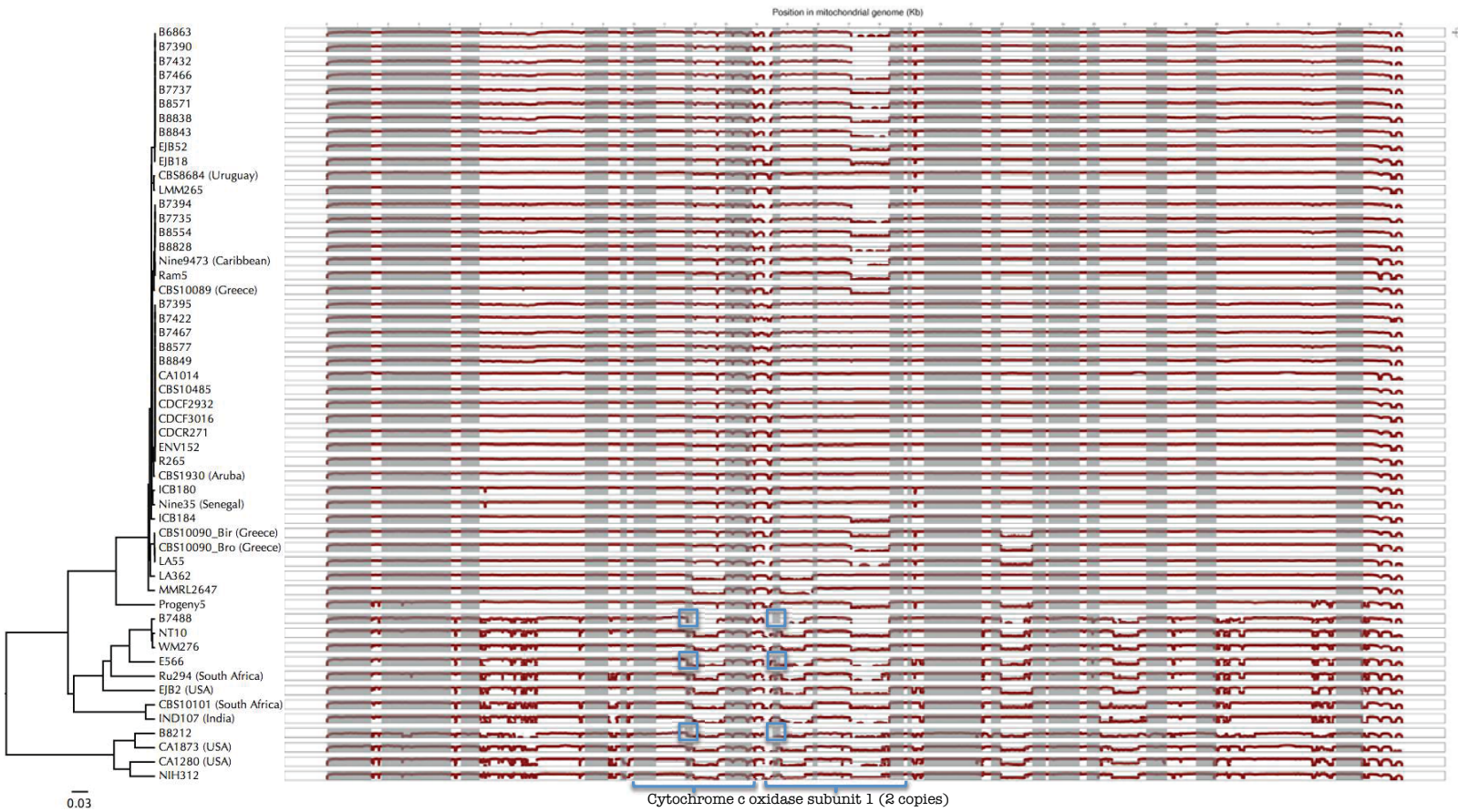

Supplement: Figure S4 — Average depth of read coverage over the mitochondrial genome for all 53 isolates. Isolates are ordered according to a tree constructed from variant sites from the mitochondrial sequences of all 53 isolates (aligned with R265), and gray boxes overlie each of the predicted R265 genes. Two copies of cytochrome c oxidase subunit 1 are predicted in R265 which are potentially absent in at least three isolates (shown by blue boxes). Depth of coverage reveals frequent intron gain/loss. Download [file mbo004152446sf4.pdf]
