# Supplementary material for: Genome Evolution and Innovation across the Four Major Lineages of Cryptococcus gattii
Source: mBio. 2015 Sep 1;6(5):e00868-15. doi: 10.1128/mBio.00868-15 (PMC4556806; doi:10.1128/mBio.00868-15)
Supplement: Figure S2 — Support of syntenic block (SB) fusion by read coverage. (A) Across region of SB2-SB3 fusion, read alignments with CA1014 were visualized using the Integrated Genomics Viewer to examine the fusion of SB2 (length, 1,124,359 bases) and SB3 identified in VGIIa CA1014, VGIIb Ram5, and MMRL2647. A drop in coverage near the fusion site is observed in all VGI, VGIII, and VGIV isolates. The single drop in VGII isolate MMRL2647 was due to a small deletion in this strain (with spanning reads). (B) Aneuploidy across the genomes was identified using the normalized depth of read coverage over each R265 supercontig for all 53 isolates and was summarized using 10-kb nonoverlapping sliding windows. Aneuploidy can be seen across supercontig 13 in VGII isolate B8828 and supercontig 11 in VGIII isolate CA1280. A large intrachromosomal duplication/expansion can also be seen in the middle of supercontig 1 for VGII isolate LA55. The start of supercontig 18 is the R265 MATα locus, which is absent for the MATa-containing VGII isolates CBS10090, LA55, and CBS1930; VGI E566; and VGIII CA1873. Download [file mbo004152446sf2.pdf]

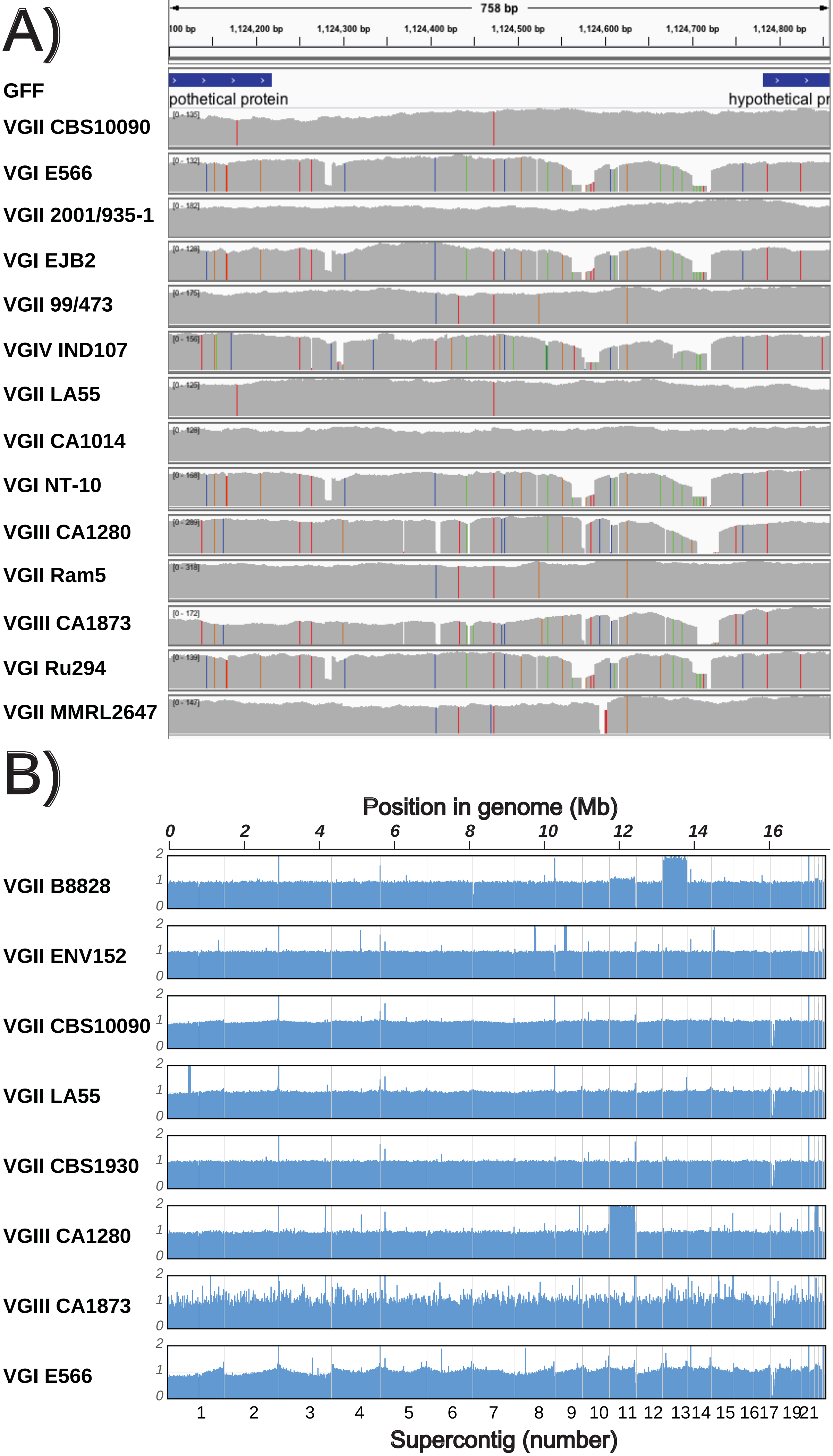
A)
758 bp
100 bp
1,124,200 bp
1,124,300 bp
1,124,400 bp
1,124,500 bp
1,124,600 bp
1,124,700 bp
1,124,800 bp
GFF
pothetical protein
hypothetical pr
VGII CBS10090
VGI E566
VGII 2001/935-1
VGI EJB2
VGII 99/473
VGIV IND107
VGII LA55
VGII CA1014
VGI NT-10
VGIII CA1280
VGII Ram5
VGIII CA1873
VGI Ru294
VGII MMRL2647
B)
Position in genome (Mb)
0
2
4
6
8
10
12
14
16
VGII B8828
VGII ENV152
VGII CBS10090
VGII LA55
VGII CBS1930
VGIII CA1280
VGIII CA1873
VGI E566
1
2
3
4
5
6
7
8
9
10
11
12
1314
15
1617
1921
Supercontig (number)
