# Supplementary material for: Genome Evolution and Innovation across the Four Major Lineages of Cryptococcus gattii
Source: mBio. 2015 Sep 1;6(5):e00868-15. doi: 10.1128/mBio.00868-15 (PMC4556806; doi:10.1128/mBio.00868-15)
Supplement: Figure S3 — Genome-wide variation in θ, Weir’s formulation of Wright’s fixation index (FST), on pairwise comparisons in each lineage. For comparison of isolates between each VG group, θ was calculated across window lengths of 10 kb. The lower FST at the start of supercontig 18 shows where the MATα locus is. Below the nonoverlapping windows, mean pairwise FST values from all nuclear supercontigs or the mitochondrial genome are shown. Download [file mbo004152446sf3.pdf]

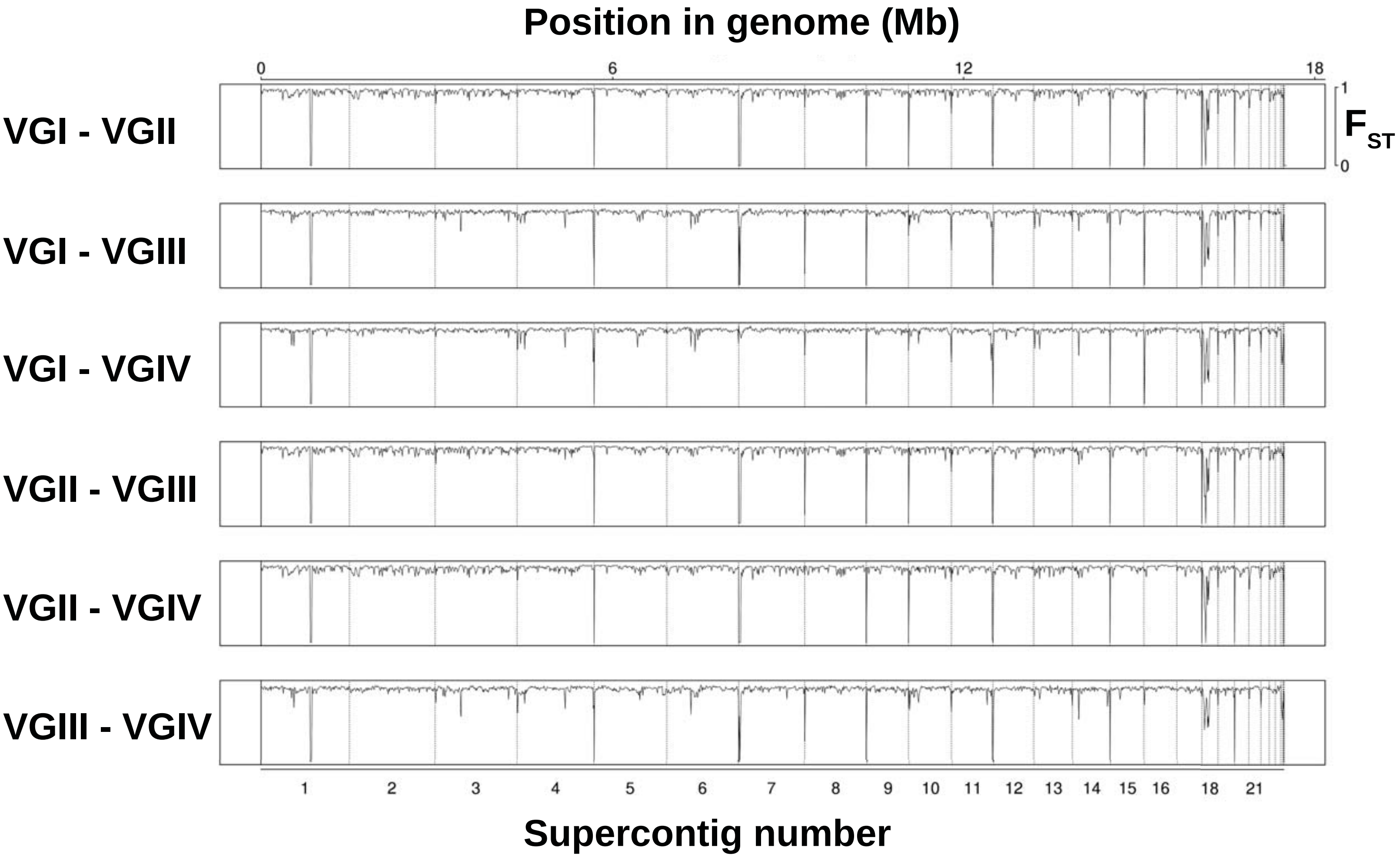

Nuclear F_{ST}

	VGI	VGII	VGIII	VGIV
VGI				
VGII	0.909			
VGIII	0.885	0.904		
VGIV	0.894	0.907	0.885	

Mitochondrial F_{ST}

	VGI	VGII	VGIII	VGIV
VGI				
VGII	0.907			
VGIII	0.642	0.894		
VGIV	0.672	0.95	0.658	
