# Supplementary material for: Genome Evolution and Innovation across the Four Major Lineages of Cryptococcus gattii
Source: mBio. 2015 Sep 1;6(5):e00868-15. doi: 10.1128/mBio.00868-15 (PMC4556806; doi:10.1128/mBio.00868-15)
Supplement: Table S1 — Repeat classification in de novo assemblies from 16 C. gattii genomes. Ranges of values obtained from RepeatModeler elements detected using RepeatMasker (number of elements, length occupied [bp], and percentage of genomes) are provided for each of the four lineages. LINEs, long interspersed elements; SINEs, short interspersed elements; LTR, long terminal repeats. [file mbo004152446st1.pdf]

	VGI	VGII	VGIII	VGIV
total length (nt)	17.67Mb-18.37Mb	17.36Mb-17.57Mb	17.44Mb-17.59Mb	17.65Mb
total length exc. Ns	17.53Mb-18.36Mb	17.23Mb-17.39Mb	17.39Mb-17.49Mb	17.56Mb
GC% level (%)	47.88-47.91%	47.82-47.85%	48.01%	47.97%
Number of SINEs	15-18	0-19	0	0
SINEs (Kb)	1.6Kb-1.9Kb	0-2.2Kb	0	0
SINEs (%)	0.01%	0-0.01%	0	0
Number of LINEs	37-89	0-27	18-30	0
LINEs (Kb)	12Kb-29Kb	0-8.8Kb	5.8Kb-7.6Kb	0
LINEs (%)	0.07-0.16%	0-0.05%	0.03-0.04%	0
Number of LTR elements	190-326	190-254	232-240	264
LTRs (kb)	44Kb-225Kb	45Kb-108Kb	62Kb-79Kb	90Kb
LTRs (%)	0.25-1.22%	0.26-0.62%	0.35%-0.45%	0.51%
Number of DNA elements	29-86	0-28	35-39	31
DNA elements (Kb)	13Kb-69Kb	0-15Kb	26Kb	20Kb
DNA elements (%)	0.07-0.38%	0-0.08%	0.15%	0.11%
Number of Unclassified repeats	261-351	139-206	222-241	175
Unclassified (Kb)	76Kb-148Kb	28Kb-50Kb	53Kb-57Kb	30Kb
Unclassified (%)	0.34-0.8%	0.16-0.29%	0.31-0.33%	0.17%
Number of ribosomal RNA	0-32	0	0	0
ribosomal RNAs (Mb)	0-0.4Mb	0	0	0
ribosomal RNAs (%)	0-2.22%	0	0	0
Number of simple repeats	827-865	687-764	716-720	752
Simple repeats (Kb)	36Kb-43Kb	30Kb-38Kb	33Kb-34Kb	35Kb
Simple repeats (%)	0.2-0.25%	0.17-0.22%	0.19%	0.20%
Number of low complexity repeats	915-942	808-846	847-863	855
Low complexity repeats (Kb)	58Kb-60Kb	52Kb-55Kb	55Kb-56Kb	55Kb
Low complexity repeats (%)	0.32-0.33%	0.3-0.31%	0.31-0.32%	0.31%
