# Supplementary material for: Genome Evolution and Innovation across the Four Major Lineages of Cryptococcus gattii
Source: mBio. 2015 Sep 1;6(5):e00868-15. doi: 10.1128/mBio.00868-15 (PMC4556806; doi:10.1128/mBio.00868-15)
Supplement: Table S2 — Pairwise comparisons from each of the 16 nuclear genome assemblies from BLASTz/Threaded Blockset Aligner (TBA) alignments. For each pairwise comparison, the number of blocks, total lengths of all blocks in parentheses, the number of matches (M), the number of mismatches (MM), and the number of gaps are shown. [file mbo004152446st2.pdf]

		VGI<br>WM276	VGI<br>E566	VGI<br>EJB2	VGI<br>NT10	VGI<br>RU294	VGIII<br>CA1280	VGIII<br>CA1873	VGIV<br>IND107	VGII<br>CBS10090	VGII<br>LA55	VGII<br>RAM5	VGII<br>99/473	VGII<br>CA1014	VGII<br>R265	VGII<br>2001/935-1
VGI	WM276															
VGI	E566	5484 (17.96 Mb)<br>M. 17.49 Mb (97%)<br>MM. 28.1 Kb<br>Gaps 442 Kb														
VGI	EJB2	5026 (17.88 Mb)<br>M. 17.43 Mb (97%)<br>MM. 16.0 Kb<br>Gaps 432 Kb	4604 (17.81 Mb)<br>M. 17.35 Mb (97%)<br>MM. 28.5 Kb<br>Gaps 430 Kb													
VGI	NT10	6042 (18.28 Mb)<br>M. 17.82 Mb (97%)<br>MM. 19.0 Kb<br>Gaps 443 Kb	5107 (17.89 Mb)<br>M. 17.42 Mb (97%)<br>MM. 31.8 Kb<br>Gaps 438 Kb	4849 (17.85 Mb)<br>M. 17.40 Mb (98%)<br>MM. 16.7 Kb<br>Gaps 428 Kb												
VGI	RU294	5265 (17.94 Mb)<br>M. 17.38 Mb (97%)<br>MM. 99.8 Kb<br>Gaps 457 Kb	4613 (17.80 Mb)<br>M. 17.23 Mb (97%)<br>MM. 109 Kb<br>Gaps 452 Kb	4447 (17.77 Mb)<br>M. 17.23 Mb (97%)<br>MM. 94.0 Kb<br>Gaps 444 Kb	4878 (17.84 Mb)<br>M. 17.30 Mb (97%)<br>MM. 98.3 Kb<br>Gaps 451 Kb											
VGIII	CA1280	3970 (17.53 Mb)<br>M. 16.28 Mb (93%)<br>MM. 656 Kb<br>Gaps 586 Kb	3451 (17.42 Mb)<br>M. 16.19 Mb (93%)<br>MM. 656 Kb<br>Gaps 579 Kb	3374 (17.43 Mb)<br>M. 16.21 Mb (93%)<br>MM. 645 Kb<br>Gaps 571 Kb	3669 (17.47 Mb)<br>M. 16.23 Mb (93%)<br>MM. 651 Kb<br>Gaps 580 Kb	3511 (17.45 Mb)<br>M. 16.22 Mb (93%)<br>MM. 650 Kb<br>Gaps 578 Kb										
VGIII	CA1873	4108 (17.48 Mb)<br>M. 16.25 Mb (93%)<br>MM. 653 Kb<br>Gaps 583 Kb	3584 (17.41 Mb)<br>M. 16.20 Mb (93%)<br>MM. 635 Kb<br>Gaps 576 Kb	3455 (17.38 Mb)<br>M. 16.17 Mb (93%)<br>MM. 641 Kb<br>Gaps 567 Kb	3796 (17.43 Mb)<br>M. 16.21 Mb (93%)<br>MM. 648 Kb<br>Gaps 576 Kb	3629 (17.40 Mb)<br>M. 16.19 Mb (93%)<br>MM. 645 Kb<br>Gaps 574 Kb	3601 (17.38 Mb)<br>M. 16.83 Mb (97%)<br>MM. 96.7 Kb<br>Gaps 459 Kb									
VGIV	IND107	4276 (17.60 Mb)<br>M. 16.29 Mb (93%)<br>MM. 713 Kb<br>Gaps 595 Kb	3672 (17.50 Mb)<br>M. 16.20 Mb (93%)<br>MM. 715 Kb<br>Gaps 587 Kb	3536 (17.48 Mb)<br>M. 16.20 Mb (93%)<br>MM. 701 Kb<br>Gaps 579 Kb	3887 (17.54 Mb)<br>M. 16.25 Mb (93%)<br>MM. 708 Kb<br>Gaps 588 Kb	3777 (17.54 Mb)<br>M. 16.25 Mb (93%)<br>MM. 707 Kb<br>Gaps 587 Kb	3233 (17.32 Mb)<br>M. 16.04 Mb (93%)<br>MM. 708 Kb<br>Gaps 575 Kb	3321 (17.28 Mb)<br>M. 16.00 Mb (93%)<br>MM. 705 Kb<br>Gaps 571 Kb								
VGII	CBS10090	3717 (17.27 Mb)<br>M. 15.75 Mb (91%)<br>MM. 913 Kb<br>Gaps 611 Kb	3147 (17.21 Mb)<br>M. 15.71 Mb (91%)<br>MM. 896 Kb<br>Gaps 603 Kb	3005 (17.18 Mb)<br>M. 15.68 Mb (91%)<br>MM. 901 Kb<br>Gaps 596 Kb	3317 (17.22 Mb)<br>M. 15.71 Mb (91%)<br>MM. 907 Kb<br>Gaps 603 Kb	3154 (17.21 Mb)<br>M. 15.70 Mb (91%)<br>MM. 906 Kb<br>Gaps 601 Kb	2738 (17.06 Mb)<br>M. 15.62 Mb (92%)<br>MM. 862 Kb<br>Gaps 578 Kb	2883 (17.05 Mb)<br>M. 15.64 Mb (92%)<br>MM. 840 Kb<br>Gaps 575 Kb	2937 (17.12 Mb)<br>M. 15.64 Mb (91%)<br>MM. 899 Kb<br>Gaps 589 Kb							
VGII	LA55	3604 (17.34 Mb)<br>M. 15.81 Mb (91%)<br>MM. 916 Kb<br>Gaps 611 Kb	3089 (17.28 Mb)<br>M. 15.77 Mb (91%)<br>MM. 899 Kb<br>Gaps 604 Kb	2954 (17.25 Mb)<br>M. 15.75 Mb (91%)<br>MM. 904 Kb<br>Gaps 598 Kb	3250 (17.29 Mb)<br>M. 15.77 Mb (91%)<br>MM. 910 Kb<br>Gaps 603 Kb	3077 (17.28 Mb)<br>M. 15.77 Mb (91%)<br>MM. 909 Kb<br>Gaps 602 Kb	2697 (17.13 Mb)<br>M. 15.69 Mb (92%)<br>MM. 865 Kb<br>Gaps 579 Kb	2815 (17.11 Mb)<br>M. 15.70 Mb (92%)<br>MM. 842 Kb<br>Gaps 575 Kb	2893 (17.20 Mb)<br>M. 15.70 Mb (91%)<br>MM. 903 Kb<br>Gaps 589 Kb	3341 (17.22 Mb)<br>M. 16.83 Mb (98%)<br>MM. 1.2 Kb<br>Gaps 395 Kb						
VGII	RAM5	3690 (17.34 Mb)<br>M. 15.82 Mb (91%)<br>MM. 906 Kb<br>Gaps 612 Kb	3112 (17.26 Mb)<br>M. 15.74 Mb (91%)<br>MM. 907 Kb<br>Gaps 605 Kb	3020 (17.26 Mb)<br>M. 15.76 Mb (91%)<br>MM. 895 Kb<br>Gaps 599 Kb	3298 (17.29 Mb)<br>M. 15.78 Mb (91%)<br>MM. 901 Kb<br>Gaps 605 Kb	3142 (17.28 Mb)<br>M. 15.78 Mb (91%)<br>MM. 900 Kb<br>Gaps 603 Kb	2789 (17.14 Mb)<br>M. 15.71 Mb (92%)<br>MM. 856 Kb<br>Gaps 580 Kb	2858 (17.12 Mb)<br>M. 15.69 Mb (92%)<br>MM. 852 Kb<br>Gaps 577 Kb	2972 (17.20 Mb)<br>M. 15.72 Mb (91%)<br>MM. 894 Kb<br>Gaps 592 Kb	3251 (17.18 Mb)<br>M. 16.71 Mb (97%)<br>MM. 64.6 Kb<br>Gaps 405 Kb	3284 (17.26 Mb)<br>M. 16.79 Mb (97%)<br>MM. 64.2 Kb<br>Gaps 407 Kb					
VGII	99/473	3536 (17.30 Mb)<br>M. 15.79 Mb (91%)<br>MM. 903 Kb<br>Gaps 607 Kb	2997 (17.22 Mb)<br>M. 15.71 Mb (91%)<br>MM. 904 Kb<br>Gaps 600 Kb	2920 (17.22 Mb)<br>M. 15.74 Mb (91%)<br>MM. 892 Kb<br>Gaps 595 Kb	3203 (17.25 Mb)<br>M. 15.75 Mb (91%)<br>MM. 898 Kb<br>Gaps 600 Kb	3038 (17.24 Mb)<br>M. 15.75 Mb (91%)<br>MM. 896 Kb<br>Gaps 599 Kb	2679 (17.11 Mb)<br>M. 15.68 Mb (92%)<br>MM. 854 Kb<br>Gaps 576 Kb	2728 (17.06 Mb)<br>M. 15.64 Mb (92%)<br>MM. 848 Kb<br>Gaps 572 Kb	2858 (17.17 Mb)<br>M. 15.69 Mb (91%)<br>MM. 891 Kb<br>Gaps 587 Kb	3143 (17.20 Mb)<br>M. 16.73 Mb (97%)<br>MM. 64.3 Kb<br>Gaps 403 Kb	3168 (17.22 Mb)<br>M. 16.75 Mb (97%)<br>MM. 63.7 Kb<br>Gaps 404 Kb	3326 (17.25 Mb)<br>M. 16.85 Mb (98%)<br>MM. 757<br>Gaps 395 Kb				
VGII	CA1014	3706 (17.39 Mb)<br>M. 15.86 Mb (91%)<br>MM. 910 Kb<br>Gaps 617 Kb	3196 (17.31 Mb)<br>M. 15.79 Mb (91%)<br>MM. 911 Kb<br>Gaps 610 Kb	3071 (17.31 Mb)<br>M. 15.81 Mb (91%)<br>MM. 898 Kb<br>Gaps 604 Kb	3363 (17.34 Mb)<br>M. 15.82 Mb (91%)<br>MM. 904 Kb<br>Gaps 610 Kb	3202 (17.33 Mb)<br>M. 15.82 Mb (91%)<br>MM. 903 Kb<br>Gaps 608 Kb	2827 (17.18 Mb)<br>M. 15.74 Mb (92%)<br>MM. 858 Kb<br>Gaps 585 Kb	2874 (17.14 Mb)<br>M. 15.70 Mb (92%)<br>MM. 854 Kb<br>Gaps 581 Kb	3018 (17.25 Mb)<br>M. 15.75 Mb (91%)<br>MM. 897 Kb<br>Gaps 596 Kb	3205 (17.22 Mb)<br>M. 16.74 Mb (97%)<br>MM. 67.4 Kb<br>Gaps 410 Kb	3251 (17.30 Mb)<br>M. 16.82 Mb (97%)<br>MM. 66.6 Kb<br>Gaps 412 Kb	3304 (17.30 Mb)<br>M. 16.83 Mb (97%)<br>MM. 56.9 Kb<br>Gaps 413 Kb	3193 (17.26 Mb)<br>M. 16.80 Mb (97%)<br>MM. 56.2 Kb<br>Gaps 409 Kb			
VGII	R265	3557 (17.17 Mb)<br>M. 15.68 Mb (91%)<br>MM. 896 Kb<br>Gaps 601 Kb	2927 (17.08 Mb)<br>M. 15.59 Mb (91%)<br>MM. 896 Kb<br>Gaps 593 Kb	2855 (17.09 Mb)<br>M. 15.62 Mb (91%)<br>MM. 884 Kb<br>Gaps 588 Kb	3162 (17.12 Mb)<br>M. 15.63 Mb (91%)<br>MM. 890 Kb<br>Gaps 594 Kb	2965 (17.11 Mb)<br>M. 15.63 Mb (91%)<br>MM. 889 Kb<br>Gaps 591 Kb	2605 (16.97 Mb)<br>M. 15.56 Mb (92%)<br>MM. 845 Kb<br>Gaps 568 Kb	2649 (16.92 Mb)<br>M. 15.51 Mb (92%)<br>MM. 841 Kb<br>Gaps 564 Kb	2795 (17.03 Mb)<br>M. 15.57 Mb (91%)<br>MM. 883 Kb<br>Gaps 580 Kb	2972 (17.00 Mb)<br>M. 16.53 Mb (97%)<br>MM. 67.1 Kb<br>Gaps 400 Kb	2999 (17.08 Mb)<br>M. 16.61 Mb (97%)<br>MM. 66.2 Kb<br>Gaps 401 Kb	3055 (17.08 Mb)<br>M. 16.62 Mb (97%)<br>MM. 56.5 Kb<br>Gaps 403 Kb	2973 (17.05 Mb)<br>M. 16.59 Mb (97%)<br>MM. 55.7 Kb<br>Gaps 400 Kb	3263 (17.15 Mb)<br>M. 16.75 Mb (98%)<br>MM. 1.1 Kb<br>Gaps 396 Kb		
VGII	2001/935-1	3649 (17.39 Mb)<br>M. 15.87 Mb (91%)<br>MM. 909 Kb<br>Gaps 618 Kb	3154 (17.31 Mb)<br>M. 15.79 Mb (91%)<br>MM. 910 Kb<br>Gaps 611 Kb	3031 (17.31 Mb)<br>M. 15.81 Mb (91%)<br>MM. 898 Kb<br>Gaps 605 Kb	3329 (17.34 Mb)<br>M. 15.83 Mb (91%)<br>MM. 904 Kb<br>Gaps 611 Kb	3152 (17.33 Mb)<br>M. 15.82 Mb (91%)<br>MM. 902 Kb<br>Gaps 609 Kb	2827 (17.20 Mb)<br>M. 15.75 Mb (92%)<br>MM. 858 Kb<br>Gaps 586 Kb	2875 (17.15 Mb)<br>M. 15.71 Mb (92%)<br>MM. 853 Kb<br>Gaps 582 Kb	2983 (17.24 Mb)<br>M. 15.76 Mb (91%)<br>MM. 895 Kb<br>Gaps 593 Kb	3225 (17.22 Mb)<br>M. 16.73 Mb (97%)<br>MM. 73.4 Kb<br>Gaps 414 Kb	3300 (17.30 Mb)<br>M. 16.81 Mb (97%)<br>MM. 73.2 Kb<br>Gaps 416 Kb	3331 (17.31 Mb)<br>M. 16.83 Mb (97%)<br>MM. 63.4 Kb<br>Gaps 416 Kb	3199 (17.27 Mb)<br>M. 16.79 Mb (97%)<br>MM. 63.2 Kb<br>Gaps 413 Kb	3392 (17.35 Mb)<br>M. 16.87 Mb (97%)<br>MM. 62.2 Kb<br>Gaps 417 Kb	3084 (17.12 Mb)<br>M. 16.66 Mb (97%)<br>MM. 61.7 Kb<br>Gaps 406 Kb	
VGII	MMRL2647	3633 (17.36 Mb)<br>M. 15.84 Mb (91%)<br>MM. 907 Kb<br>Gaps 618 Kb	3093 (17.28 Mb)<br>M. 15.76 Mb (91%)<br>MM. 908 Kb<br>Gaps 611 Kb	3008 (17.29 Mb)<br>M. 15.78 Mb (91%)<br>MM. 897 Kb<br>Gaps 605 Kb	3292 (17.31 Mb)<br>M. 15.80 Mb (91%)<br>MM. 902 Kb<br>Gaps 611 Kb	3122 (17.31 Mb)<br>M. 15.80 Mb (91%)<br>MM. 901 Kb<br>Gaps 608 Kb	2833 (17.17 Mb)<br>M. 15.73 Mb (92%)<br>MM. 858 Kb<br>Gaps 586 Kb	2881 (17.15 Mb)<br>M. 15.71 Mb (92%)<br>MM. 855 Kb<br>Gaps 582 Kb	3013 (17.23 Mb)<br>M. 15.74 Mb (91%)<br>MM. 895 Kb<br>Gaps 591 Kb	3152 (17.19 Mb)<br>M. 16.67 Mb (97%)<br>MM. 100 Kb<br>Gaps 420 Kb	3205 (17.27 Mb)<br>M. 16.75 Mb (97%)<br>MM. 99.8 Kb<br>Gaps 423 Kb	3293 (17.30 Mb)<br>M. 16.78 Mb (97%)<br>MM. 91.1 Kb<br>Gaps 424 Kb	3149 (17.24 Mb)<br>M. 16.73 Mb (97%)<br>MM. 90.5 Kb<br>Gaps 419 Kb	3294 (17.32 Mb)<br>M. 16.80 Mb (97%)<br>MM. 89.2 Kb<br>Gaps 425 Kb	3072 (17.10 Mb)<br>M. 16.60 Mb (97%)<br>MM. 88.2 Kb<br>Gaps 414 Kb	3376 (17.34 Mb)<br>M. 16.83 (97%)<br>MM. 87.5 Kb<br>Gaps 423 Kb
