# Supplementary material for: Genome Evolution and Innovation across the Four Major Lineages of Cryptococcus gattii
Source: mBio. 2015 Sep 1;6(5):e00868-15. doi: 10.1128/mBio.00868-15 (PMC4556806; doi:10.1128/mBio.00868-15)
Supplement: Table S3 — Fifteen large (>100-kb) potential chromosomal rearrangements were identified among the 16 genomes shown in Fig. S2, S3, and S4. Here, each C. gattii genome is divided into syntenic blocks (SB), with each row showing a discrepancy (highlighted in red), and described in the final 4 columns (the isolate, inter-/intrachromosome changes, abbreviated description, and size in kilobases). In total, these changes cover 7.3 Mb of sequence/2.6% of all the 16 genomes. hf, highly fragmented (>4 supercontigs per SB block); S, start of supercontig; E, end of supercontig; NA, nonapplicable; T, translocation; I, inversion; F, fused. *, SB9 does not have any discrepancies between these isolates but is included to complete the comparisons of each of the genomes. [file mbo004152446st3.pdf]

Syntenic Block (SB)	VGI					VGIII		VGIV	VGII								Change in isolate(s)	Inter/ Intra-	Desc.	Size (Kb)
	WM276	E566	EJB2	NT10	Ru294	CA1280	CA1873	IND107	CBS 10090	LA55	Ram5	99/473	CA1014	R265	2001/ 935-1	MMRL 2647				
1	b-	26+, 25+, 23-, 03+	h.f.	h.f.	31-, 35+, 13-, 04+	20+, 01-	22+, 16+, 05-	22+, 15+, 04-, 27-	18+,-3-	h.f.	20-, 17+, 07+	h.f.	18-, 03+	24+, 22-, 01+	17-, 13+, **S02+**	20+, 12+, 07-	2001/935-1	NA	F.SB2	1,094
2	k+	06+	h.f.	h.f.	06-	*07+*	07-	20-	06-	59+, 03+, 57-, 53+	**S01-**	49+, 01+, 37+	**S02-**	07+	E02+	**S01-**	Ram5, CA1014, MMRL2647	NA	F.SB3	1,115
2	k+	06+	h.f.	h.f.	06-	07+	**07-**	20-	**06-**	59+, 03+, 57-, 53+	S01-	49+, 01+, 37+	S02-	07+	E02+	S01-	IND107	Inter	T.SB1	454
3	c+	13+, 27+, 02-	h.f.	h.f.	05+, 03-	**06+**, 04+	25-, 04-	**06+**,05+	07+	60+, 02+	E01-	32+, 14+, 15+	E02-, 19+	08+	08-	E01-	CA1873	Inter	I.T.SB5	696
4	m+	15-	h.f.	h.f.	34+, 15+	30-, 14-	27-, 14+	30-,14+	13+	51+, 13+, 43+	14+	11+	12+	13+	11+	**S03+**	MMRL2647	NA	F.SB5	990
5	a-	**01-**,09+	h.f.	h.f.	h.f.	18+, 05+, 11+, 27+	01+, 11-	19+, 07+, 11-	02-	h.f.	03-,12-	h.f.	06+, 11+	03+, 16-	03+	E03+, 17+, 10+	WM276	Inter	T.SB11	113
6	j-,f-	24-, 32+, 11-, 18+	h.f.	h.f.	**19-**, 16-, 30+, 17-	24-, 16+, 31-, 15+	06+, 18+	08+,17+	04+	09+, 23+, 10+	08+,18+	38+, 07+, 64+, 16+	08+, 16-	04+	04+	04-	NT10	Intra	T.	108
7	e-	14+,08+	h.f.	h.f.	25+, 22+, **02+**	13-,**10+**	*15+, 10+*	13-,10-	05+	07+, 05+, 45-	16-,11+	09+,05+	04+	15+, 09+	05-	05-	Ru294	NA	F.SB8	698
8	**h+**	**21+,07+**	h.f.	h.f.	24+,12-	17+, 08-, 21+	08+, 13+	03-	10-,16+	h.f.	05-	h.f.	09-,17+	06-	07+	06+	WM276	Intra	F.	406
8	h+	21+,07+	h.f.	h.f.	24+,12-	17+, **08-**, 21+	**08+**, 13+	03-	10-,16+	h.f.	05-	h.f.	09-,17+	06-	07+	06+	CA1280 CA1873	Inter	T.SB10	142
8	h+	21+,07+	h.f.	h.f.	24+,12-	**17+**, 08-, 21+	08+, 13+	03-	10-,16+	h.f.	05-	h.f.	09-,17+	06-	07+	06+	CA1280	Intra	T.	410
9*	g+	20+,04+	h.f.	h.f.	20+, 07-, 36+	03+,43+	03-	02+	15-,09+	14-, 15-, 11-	04-	21-, 53-, 02+	05+	05-	06+	16+, 08+	NA	NA	NA	NA
10	i+	29+, 17+, 30+, 31-	h.f.	h.f.	08+,27-	09+,26+	09+, 24+	09-, 23+, 25-	08+,**12-**	h.f.	06+,10-	h.f.	07+, 20-, 13+	18-, 20+, 17+, 10+	09+, 18+, 10+	09-, 18-, 21+, 11-	IND107	Inter	T.SB3	541
10	i+	29+, 17+, 30+, **31-**	h.f.	h.f.	08+,27-	09+,26+	09+, 24+	09-, 23+, 25-	08+,**12-**	h.f.	06+,10-	h.f.	07+, 20-, 13+	18-, 20+, 17+, 10+	09+, 18+, 10+	09-, 18-, 21+, 11-	WM276	Inter	T.SB2	180
11	n+	16-, **28-**	h.f.	h.f.	28-, 14+, 32-, 26+	h.f.	17+, 26-, 21+, 23-	h.f.	14+, 20-, 17-, 19+	h.f.	15-,19+	h.f.	14-, 21+, 15+	14-, 19-, 21+	12+,14-	13+, 22+, 15+	WM276	Inter	T.SB5	226
11	n+	16-,28-	h.f.	h.f.	28-, 14+, 32-, 26+	**h.f.**	17+, 26-, 21+, 23-	h.f.	14+, 20-, 17-, 19+	h.f.	15-,19+	h.f.	14-, 21+, 15+	14-, 19-, 21+	12+,14-	13+, 22+, 15+	Ru294	Inter	T.SB6	187
