# Supplementary material for: Genome Evolution and Innovation across the Four Major Lineages of Cryptococcus gattii
Source: mBio. 2015 Sep 1;6(5):e00868-15. doi: 10.1128/mBio.00868-15 (PMC4556806; doi:10.1128/mBio.00868-15)
Supplement: Table S4 — Alignments using BWA-MEM and variant calls using GATK UnifiedGenotyper for 52 isolates of C. gattii. Isolates were sequenced by the Broad Institute (BI), the Centers for Disease Control and Prevention (CDC) with collaboration from Joe Heitman’s lab, or Imperial College London/Birmingham University (ICL/BU). Red signifies predicted MAT locus based on depth of coverage. Mb, megabase; *, in vitro cross between NIH312 and CBS10090. [file mbo004152446st4.pdf]

Lineage	Isolate	Mating Type	Sequenced by	nt aligned (Mb)	Depth	Reference	SNP	Insertion	Deletion	Breadth of coverage (%)
VGI	B7488	MATα	CDC	795	46.08	15,645,425	798,557	34,517	31,024	95.65
VGI	E566	MATa	BI	2,157	124.98	15,638,779	810,372	35,999	32,445	95.69
VGI	EJB2	MATα	BI	2,113	122.39	15,697,141	810,831	36,051	32,479	96.04
VGI	NT10	MATα	BI	2,746	159.06	15,719,710	815,617	36,409	32,877	96.20
VGI	Ru294	MATα	BI	2,644	153.17	15,720,145	816,863	36,351	32,684	96.21
VGI	WM276	MATα	ICL/BU	1,868	108.21	15,790,406	808,349	35,528	31,839	96.55
VGII	2001/935-1	MATα	BI	3,296	190.98	17,085,580	60,162	2,960	3,348	99.37
VGII	CBS10089	MATα	ICL/BU	1,013	58.67	17,112,033	57,092	2,684	3,230	99.50
VGII	CBS1930	MATa	ICL/BU	2,253	130.52	17,044,711	61,114	2,840	3,305	99.14
VGII	CBS8684	MATα	ICL/BU	5,597	324.24	17,114,271	63,033	2,980	3,516	99.55
VGII	ICB180	MATα	ICL/BU	1,561	90.44	17,089,754	61,062	2,880	3,297	99.40
VGII	ICB184	MATα	ICL/BU	1,746	101.13	17,123,653	54,504	2,666	3,116	99.55
VGII	LA362	MATα	ICL/BU	706	40.88	17,077,000	59,436	2,894	3,133	99.31
VGII	LMM265	MATα	ICL/BU	3,350	194.06	17,118,584	61,256	2,889	3,297	99.57
VGII	MMRL2647	MATα	BI	2,610	151.18	17,012,693	86,037	4,111	4,134	99.11
VGIIa	B7395	MATα	CDC/Heitman	721	41.75	17,226,553	314	75	456	99.81
VGIIa	B7422	MATα	CDC	1,742	100.92	17,220,998	304	72	459	99.77
VGIIa	B7467	MATα	CDC	714	41.34	17,214,245	327	73	459	99.74
VGIIa	B8577	MATα	CDC	767	44.43	17,225,752	301	74	456	99.80
VGIIa	B8849	MATα	CDC	999	57.89	17,227,039	347	76	459	99.81
VGIIa	CA1014	MATα	BI	2,790	161.63	17,218,992	439	83	462	99.76
VGIIa	CBS10485	MATα	ICL/BU	795	46.05	17,223,549	329	72	450	99.79
VGIIa	CDCF2932	MATα	ICL/BU	1,877	108.76	17,228,385	379	75	454	99.82
VGIIa	CDCF3016	MATα	ICL/BU	4,138	239.74	17,233,503	395	73	458	99.85
VGIIa	CDCR271	MATα	ICL/BU	1,159	67.12	17,224,184	355	69	451	99.79
VGIIa	ENV152	MATα	ICL/BU	4,409	255.43	17,231,463	389	74	458	99.84
VGIIa	R265	MATα	ICL/BU	3,226	186.89	17,231,371	316	72	454	99.83
VGIIb	99/473	MATα	BI/Heitman	3,044	176.34	17,103,318	54,715	2,666	3,129	99.44
VGIIb	B7394	MATα	CDC	334	19.33	17,065,370	53,781	2,423	2,913	99.21
VGIIb	B7735	MATα	CDC	358	20.74	17,067,492	53,931	2,363	2,870	99.22
VGIIb	B8554	MATα	CDC	1,273	73.74	17,112,805	54,350	2,589	3,064	99.49
VGIIb	B8828	MATα	CDC	608	35.20	17,082,018	53,977	2,515	3,004	99.31
VGIIb	Ram5	MATα	BI	5,795	335.74	17,079,760	55,256	2,669	3,125	99.31
VGIIc	B6863	MATα	CDC	591	34.23	17,086,002	56,568	2,629	3,193	99.35
VGIIc	B7390	MATα	CDC	579	33.56	17,081,777	56,592	2,622	3,187	99.32
VGIIc	B7432	MATα	CDC	480	27.78	17,079,167	56,529	2,578	3,171	99.08
VGIIc	B7466	MATα	CDC	773	44.80	17,089,414	56,690	2,649	3,220	99.37
VGIIc	B7737	MATα	CDC	717	41.55	17,086,741	56,640	2,663	3,232	99.35
VGIIc	B8571	MATα	CDC	851	49.31	17,092,433	56,806	2,665	3,234	99.39
VGIIc	B8838	MATα	CDC	987	57.19	17,089,062	56,817	2,673	3,227	99.37
VGIIc	B8843	MATα	CDC	1,038	60.15	17,092,316	56,812	2,679	3,232	99.39
VGIIc	EJB18	MATα	ICL/BU	1,027	59.49	17,097,937	56,807	2,666	3,227	99.42
VGIIc	EJB52	MATα	ICL/BU	1,026	59.43	17,097,403	56,965	2,681	3,228	99.42
VGIII	B8212	MATα	CDC	425	24.61	15,734,956	756,082	27,909	29,564	95.87
VGIII	CA1280	MATα	BI	4,476	259.34	15,811,930	800,977	31,747	34,097	96.63
VGIII	CA1873	MATa	BI	2,634	152.60	15,734,030	779,110	31,131	33,063	96.04
VGIII	NIH312	MATα	ICL/BU	2,999	173.76	15,895,723	796,314	31,257	33,462	97.08
VGIII	Progeny5*	MATα	ICL/BU	1,347	78.07	15,822,731	784,795	30,385	32,585	96.58
VGIIx	CBS10090_Bir	MATα	ICL/BU	669	38.78	17,037,737	55,008	2,456	3,021	99.06
VGIIx	CBS10090_Bro	MATα	BI	2,370	137.31	17,040,878	56,012	2,582	3,120	99.08
VGIIx	LA55	MATa	BI	1,698	98.35	17,038,999	55,945	2,575	3,105	99.07
VGIV	CBS10101	MATα	ICL/BU	1,398	80.97	15,799,091	810,906	33,545	32,605	96.61
VGIV	IND107	MATα	BI	2,364	136.93	15,735,430	818,105	34,433	33,584	96.30
